# Novel 3D geometry and models of the lower regions of large trees for use in carbon accounting of primary forests

**DOI:** 10.1093/aobpla/ply015

**Published:** 2018-02-28

**Authors:** Christopher Dean, Jamie B Kirkpatrick, Jon Osborn, Richard B Doyle, Nicholas B Fitzgerald, Stephen H Roxburgh

**Affiliations:** 1 School of Technology, Environments and Design, University of Tasmania, Hobart, ustralia; 2 Tasmanian Institute of Agriculture, University of Tasmania, Hobart, TAS , Australia; 3 CSIRO Land & Water, Canberra, ACT, Australia

**Keywords:** 3D, allometric, buttress, humus mound, land-use emissions, primary forest, root volume, soil carbon

## Abstract

There is high uncertainty in the contribution of land-use change to anthropogenic climate change, especially pertaining to below-ground carbon loss resulting from conversion of primary-to-secondary forest. Soil organic carbon (SOC) and coarse roots are concentrated close to tree trunks, a region usually unmeasured during soil carbon sampling. Soil carbon estimates and their variation with land-use change have not been correspondingly adjusted. Our aim was to deduce allometric equations that will allow improvement of SOC estimates and tree trunk carbon estimates, for primary forest stands that include large trees in rugged terrain. Terrestrial digital photography, photogrammetry and GIS software were used to produce 3D models of the buttresses, roots and humus mounds of large trees in primary forests dominated by *Eucalyptus regnans* in Tasmania. Models of 29, *in situ* eucalypts were made and analysed. 3D models of example eucalypt roots, logging debris, rainforest tree species, fallen trees, branches, root and trunk slices, and soil profiles were also derived. Measurements in 2D, from earlier work, of three buttress ‘logs’ were added to the data set. The 3D models had high spatial resolution. The modelling allowed checking and correction of field measurements. Tree anatomical detail was formulated, such as buttress shape, humus volume, root volume in the under-sampled zone and trunk hollow area. The allometric relationships developed link diameter at breast height and ground slope, to SOC and tree trunk carbon, the latter including a correction for senescence. These formulae can be applied to stand-level carbon accounting. The formulae allow the typically measured, inter-tree SOC to be corrected for not sampling near large trees. The 3D models developed are irreplaceable, being for increasingly rare, large trees, and they could be useful to other scientific endeavours.

## Teaser

The largest uncertainty in human’s contribution to climate change from land use is the fate of carbon that was below ground in pre-modified forests. We produced high-resolution 3D models of the rarely measured zone near the base of large, mature trees by using photogrammetry. The models led to equations linking the easy-to-measure trunk diameter and ground slope to attributes such as tree buttress shape, humus mound, wood and hollow area, and root volume. The equations can be used for carbon accounting. The 3D models are irreplaceable, being for increasingly rare, large trees, and may be useful to other scientific endeavours.

## Introduction

Uncertainty in anthropogenic carbon emissions and carbon cycle-climate feedback are the two main contributors to uncertainty in the temperature effect of climate change to 2100 (Meir *et al.* 2006). The uncertainty in carbon emissions accompanying land-use change (LUC) (which includes change in forest logging intensity; Luyssaert *et al.* 2014) remains the most uncertain emissions in the global carbon budget (Canadell *et al.* 2007). This is mostly due to uncertainty in change in soil organic carbon (SOC) and roots (Kim and Kirschbaum 2014; Luo *et al.* 2015). Fossil fuel emissions have only recently surpassed those from land use (1988(±20); Dean *et al.* 2012a). However, zero change in soil organic carbon (∆SOC) with timber harvesting was assumed in that tally (Houghton 2008), which more comprehensive work revealed as unlikely (e.g. Diochon *et al.* 2009; Zummo and Friedland 2011; Clarke *et al.* 2015; Dean *et al.* 2017). The likely change in soil carbon with land use reinforces the need to refine our knowledge of the soil carbon in primary forests.

Sampling SOC under and adjacent to large tree trunks appears to be extremely rare in temperate, boreal or tropical forests due to physical constraints (e.g. Bockheim 1977; Entry and Emmingham 1998; Fang *et al.* 2010; Dietrich 2012; Fedrigo *et al.* 2014; Schrumpf *et al.* 2014). Soil organic carbon can be more concentrated closer to trees, either through thicker humus layers or higher C concentration in the mineral soil, or both (Lutz 1960; Liski 1995; Throop and Archer 2008; Rossetti *et al.* 2015). Conversely, the coarse roots of large trees will displace soil, therefore reducing SOC per unit area to some degree in the vicinity of those trees. Tree trunks, roots and soil are often highly disturbed during some types of logging such as clearfell-and-burn, thus making soil carbon that was near coarse tree roots much more physically accessible (Ellis and Graley 1983; Rab 1996; Pennington *et al.* 2001), and in turn making the comparison of pre- and post-logging soil carbon stocks difficult. Mature trees can leave a long-term, spatial, chemical signal (imprint) in the soil (Døckersmith *et al.* 1999; Phillips and Marion 2005). However, the size of the largest trees remaining in existence has been decreasing (Herrmann 2006; Lindenmayer *et al.* 2012); therefore, the original source of some long-term imprints may be no longer measurable—another motive for studying remnant larger trees.

Improvement in the calculation of LUC effects requires determination of the stand-level effects of large trees on SOC, a prerequisite for which is the characterization of the macroscopic tree-soil interface. The trunks of large trees near where they meet the ground have only recently begun to be measured in a way that can assist calculation of carbon dynamics (e.g. Dean 2003; Chave *et al.* 2005; Dean and Roxburgh 2006; Nogueira *et al.* 2006; Ngomanda *et al.* 2012; Sillett *et al.* 2015). Moreover, the root volume near large tree trunks, which displaces SOC, has rarely been measured.

Another tree-associated feature of many temperate primary forests that is seldom measured is the humus mound. These mounds accumulate as pyramidal-shaped collections of fermentation and humus material around the base of large trees. Humus mounds are called ‘duff mounds’ in the USA (Ryan and Frandsen 1991). Large humus mounds are in part a product of the ecological integration of stemflow (of rainwater) and the facultative epiphytes (Moffett 2000); or ‘hemi-epiphytes’ (Petrie *et al.* 1929), which can include mature trees (Levia and Frost 2003; Oyarzún *et al.* 2011) **[see****Supporting Information—Fig. S2****]**. The humus mounds can be up to ‘several feet’ deep (Cremer 1962), increasing with tree age and size in the absence of fire (Ashton 1981; Bens *et al.* 2006; Penne *et al.* 2010). The humic material contributes to SOC stock, also in the mineral soil below the humus mound, transported there by canopy throughfall and stemflow (Liski 1995). Thus, the large humus mounds are possible locations of concentrated C and are relevant to carbon accounting.

The forests that are the subject of the present paper are dominated by *Eucalyptus regnans*. It is one of the tallest tree species and their primary forests have amongst the highest above-ground carbon density of any forest (Hickey *et al.* 2000; Keith *et al.* 2009; Wood *et al.* 2010; Fedrigo *et al.* 2014). Therefore, they are likely candidates for detection of any localized, individual-tree influence on soil carbon concentration. The shape of the buttress regions of mature *E. regnans* has previously been calculated using standard measuring tapes but with relatively few data for such complex curvature (Dean *et al.* 2003; Dean and Roxburgh 2006; Sillett *et al.* 2015). Above-ground biomass has been calculated from manual measurement of trunks and branches while climbing *E. regnans* (Sillett *et al.* 2015) but not for the more mature trees, which possess senescence effects such as large basal hollows or depleted crowns. Above-ground external wood volume of large *E. regnans* (with hollows) was earlier estimated by 2D remote sensing using film photography and photogrammetry (Dean 2003). The 3D surfaces of solid objects can now be reconstructed from photographs taken with digital cameras plus photogrammetric techniques, particularly the structure-from-motion multi-view-stereopsis algorithms (SfM) employed by software such as Photoscan (Agisoft 2015). Scanning LiDAR has been used to derive geometrically accurate representations of the surfaces, and the volumes or masses of the above-ground woody components and roots of trees (Calders *et al.* 2015; e.g. Gärtner *et al.* 2009; Dassot *et al.* 2012; Kaasalainen *et al.* 2014; Smith *et al.* 2014; Hackenberg *et al.* 2015). Both LiDAR and SfM may be suitable for studying individual tree biomass (Dittmann *et al.* 2017). The capacity of Photoscan for photogrammetric image processing at the scales and resolutions necessary for mathematically characterizing trees has been proven (e.g. Verhoeven 2011; Morgenroth and Gomez 2013; Jiroušek *et al.* 2014; Liang *et al.* 2014; Bauwens *et al.* 2017). The method has high metric accuracy and precision (depending on the geometry and quality of the original photography) and is suitable for trees, tree parts and ground topography measurement.

In *E. regnans* primary forest in Tasmania, Australia, we demonstrate a methodology of shape calculation for buttresses, humus mounds and roots that enables our development of allometric equations, based on simple, above-ground tree diameter measurement. These allometric equations will be designed for use in primary forest stands containing large trees, to enable rapid correction of measured SOC and to aid in the determination of the amount of carbon in the tree trunks, both at the stand level.

## Methods

### Terminology

For mature *E. regnans* trees the buttress region is anatomically complex. It starts where the lateral roots merge with the soil, which can be below 0 m on the downhill side of the tree, and extends as far as 9–18 m up the trunk where the bark changes from sub-fibrous to the smooth (‘gum’) bark (Ashton 1975). ‘Spurs’ are the ridges of the buttress which join the main trunk to the large lateral roots. ‘Flutes’ are the valleys between spurs (Julin *et al.* 1998). We define the footprint of a tree as the area inside a convex hull polygon that circumscribes where the spurs merge with the forest floor. The forest floor is the layer of material including the litter, fermentation and humus layers above the mineral soil (i.e. above the soil A horizon).

### Study site

**Figure 1.**
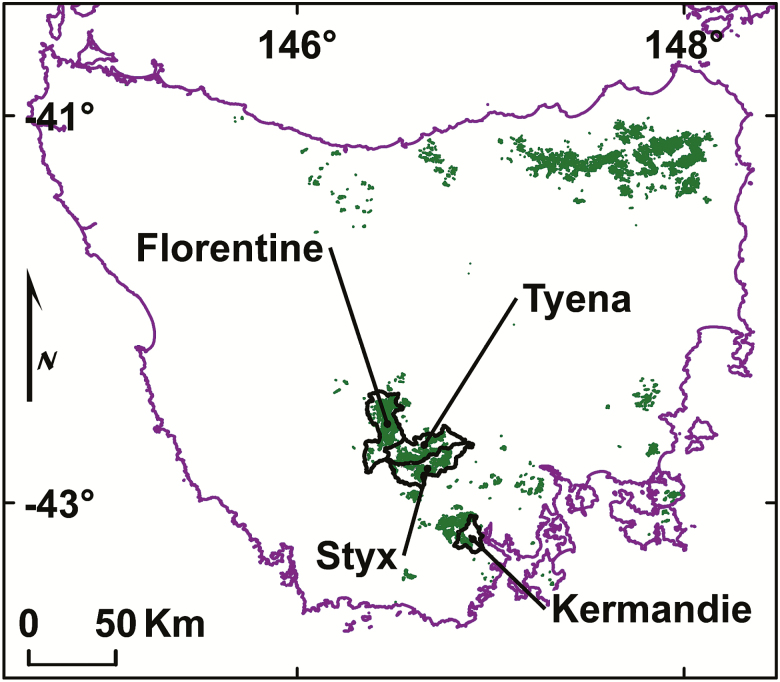
Study area. Catchments (outlined in black) in Tasmania, Australia where data were acquired. Green area shows approximate distribution of *Eucalyptus regnans*-dominated forests. Pseudo Plate Carree projection, spheroid WGS1984, lat/long coordinates.

Data were collected across an elevation range of 221–659 m in primary tall-eucalypt forests with a rainforest understorey (mixed forest) in the Styx, Tyenna, Florentine and Kermandie catchments in Tasmania, Australia (Fig. 1). Soil data from the humus mounds were collected in the Styx Valley, where annual rainfall is 1170 mm (over the period 1993–2013) with a weak winter maximum. The mean daily minimum temperature in the coldest month is 2.3 °C and the mean daily maximum in the warmest month is 22.4 °C. The soils are Cambisols (IUSS WG 2014) in the FAO classification, derived from sedimentary rocks. Google Earth© imagery was used to select sites with particular characteristics; as a navigation aid; and for locating suitable study trees pre-logging, and stumps post-logging. GIS (ArcGIS, ESRI) was used extensively, for navigation, spatial data preparation, combining data layers, cartography and spatial calculations.

### Overview of mensuration and modelling

The standard forest mensuration technique used in temperate forests was employed: tree diameter at ‘breast height’ (DBH) over bark, i.e. 1.3 m from the ground on the high side of the tree and including adjustment for stem slope (IPCC 2003). For large trees the orientation of the tape was checked with a clinometer. Diameter at breast height measurement has the benefit of universality in databases and allometric equations for biomass or carbon. Where visibility through the closed understorey of the mixed forest permitted, a clinometer and laser rangefinder were used for measuring tree height.

Incorporating tree height into allometric equations can result in inaccurate outcomes for mature trees, for example after crown loss (e.g. Biggs 1991), or crown regeneration. Diameter at breast height-based allometric equations can be inaccurate predictors if, for example, applied to trees of different fire or histories and therefore different hollow sizes (Adkins 2006), or of different senescence, to those from where the allometry was developed.

The conversion of DBH, as measured using a girth tape, to cross-sectional area assumes that the trunk is circular. Any stem non-circularity, including flutes (gaps) between spurs on the buttress, gives a falsely high value, with the difference between that and a circle being the ‘cross-sectional area deficit’ (Dean 2003; Keith *et al.* 2014). For buttressed trees, such as *E. regnans*, in place of the standard DBH measurement, Sillett *et al.* (2015) formulated a ‘functional diameter’ which was a measure of the wood cross-sectional area without any area deficit. The functional diameter potentially allows higher consistency in formulating biomass allometry, but it is still necessary to take the standard DBH measurement, as that is what can be measured most readily in the forest for a stand of trees and it allows comparison with a range of other work. The present work uses the standard DBH measurement, followed by parameterization of the cross-section.

We trialled the use of tripod-based, multiple-scan LiDAR (MS) and handheld mobile LiDAR scanning HMLS (Bauwens *et al.* 2016). The MS apparatuses used were a RIEGL VZ400 and a Faro Photon 20. The method of Raumonen *et al.* (2013) and Calders *et al.* (2015) can be used to determine accurate above-ground biomass. It uses quantitative structure models formed from surface segments of cylindrical shape, and it was verified for dry-schlerophyll eucalyptus trees up to about DBH 0.62 m in Calders *et al.* (2015) by destructive sampling. The HMLS apparatus was Zebedee (Bosse *et al.* 2012).

Our study trees were an order of magnitude wider than those studied earlier by MS, their epiphytes were sometimes larger than the trees in the earlier MS studies, and our forest stands had an order of magnitude higher basal area. Some of our study trees had large basal hollows. These attributes created complications for use of MS. For example, too many or too complicated vantage points were required: (i) to circumnavigate fluted buttresses of 6 or 7 m diameters; (ii) to see around nearby understorey vegetation; and (iii) to scan a complex, 3 m high root ball. The MS required a generator and two tripods, which were too difficult to carry over the rugged terrain with a crew of less than three. Additionally, the tripods could not be stabilized on some of the necessary vantage points such as deep debris heaps, unstable slopes and large, slippery logs. Additionally, if one had wanted to measure whole above-ground biomass, ground-based LiDAR MS could not collect data on the mid-to-upper regions of large eucalypt trees because of the closed rainforest understorey beneath them. The HMLS could cope with the rugged terrain and most of the understorey but the surface accuracy of 0.02 m (R. Zlot, CSIRO, pers. comm.) was too low for our needs. Additionally, the absence of colour information with HMLS precluded subtraction of moss and understorey to reveal only the bark on the trunk of interest. MS and HMLS data were not used further in the present study.

**Figure 2.**
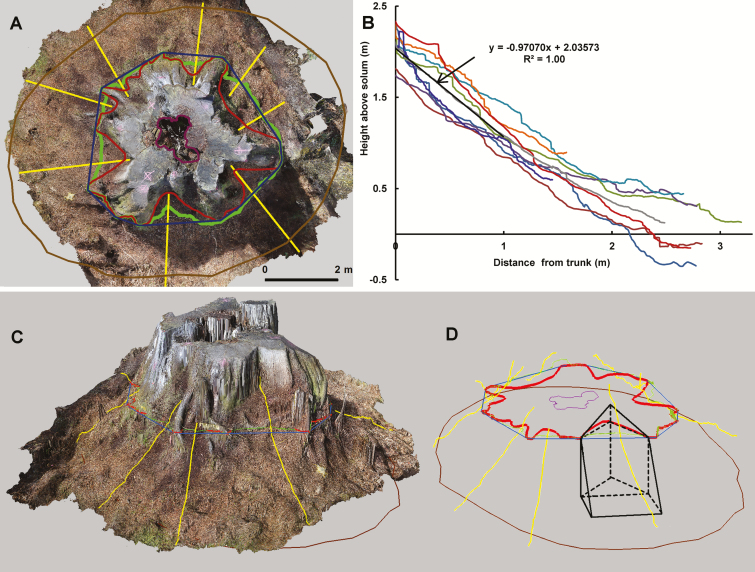
3D model from Photoscan with ArcGIS markup, showing buttress region and humus mound. *Eucalyptus regnans*, DBH = 4.95 m, shown before logging in Dean (2003) and **Supporting Information—Fig. S2E**. (A) Top view, lines: outermost = footprint, innermost = hollow, in between and convoluted = corrected 1.3 m contour, moderately convoluted = 1.3 m contour, least convoluted = convex hull at 1.3 m, radial = humus profiles. (B) Graphed humus profiles and line of best fit, (C and D) oblique view, (D) example triangular and rectangular pyramids used to calculate humus volume for a flute.

**Figure 3.**
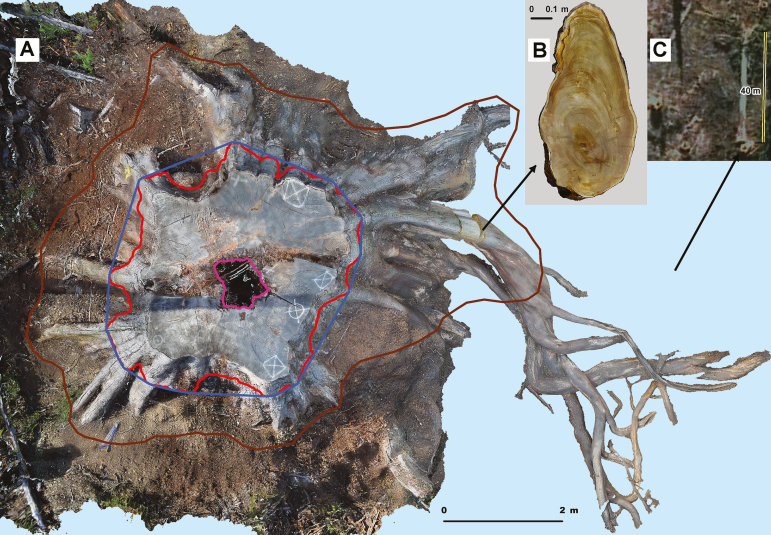
Buttress area characterization from 3D model, example 1. *Eucalyptus regnans* (DBH 4.38 m, Tyenna Valley). (A) Orthophoto created using terrestrial photography and Photoscan, top view. Red line = 1.3 m corrected contour, blue line = convex hull at 1.3 m matching DBH tape, brown line = footprint, magenta line = hollow. Humus mound removed, soil removed from one major lateral root. Coarse roots extend beyond footprint. Roots continue spiral grain of trunk and are plaited, curling around hemi-epiphytic trees and aiding host tree stability. (B) 3D model of root slice of large lateral within the footprint, ring age count = 350(±40) years. (C) Google Earth® satellite image shows felled trunk, stump and neighbouring stumps, during logging (scale bar = 40 m) (insets enlarged in **Supporting Information—Fig. S5**).

**Figure 4.**
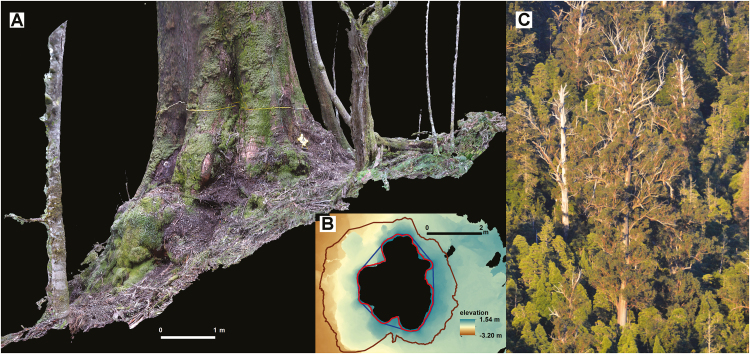
Buttress area characterization from 3D model, example 2. *Eucalyptus regnans* (DBH 3.24 m, height 64 m, Styx Valley) on steep slope (24°) with minimal humus mound: volume = 0.96 m^3^. (A) Orthophoto created using terrestrial photography and Photoscan. (B) Topography (DEM), 1.3 m corrected contour, convex hull and footprint. (C) Upper portion of tree in ‘(A)’, above neighbouring rainforest understorey, and with ample foliage and original crown.

**Figure 5.**
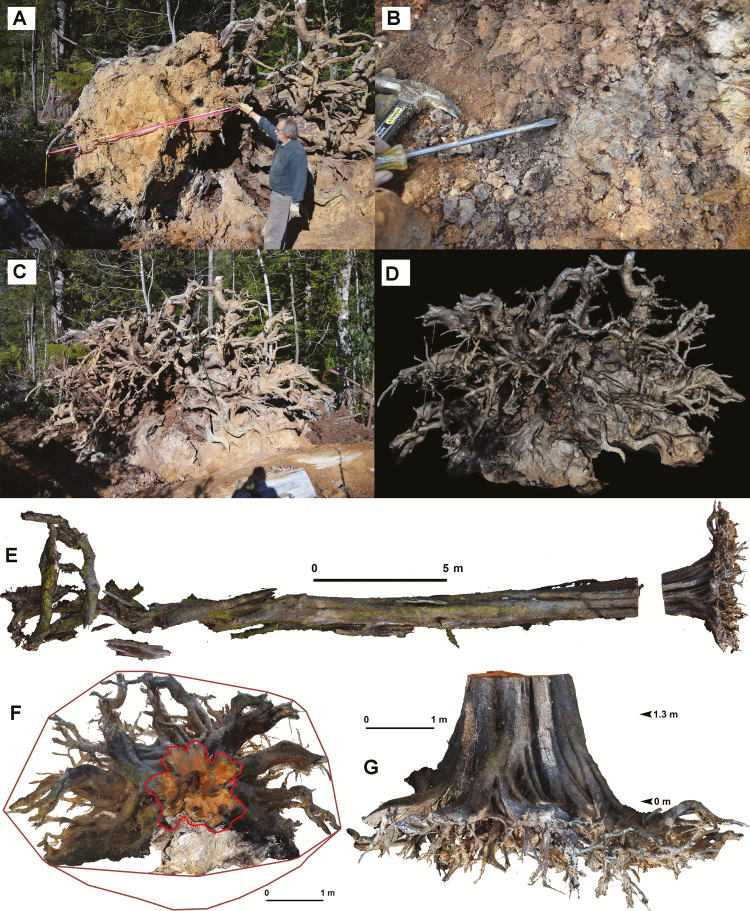
Components of geometric analysis of myrtle tree DBH 1.76 m, height 32(±4) m, pushed over during logging. Process of myrtle root excavation (A) (with Prof. Kirkpatrick) and (B); photography with scale bars for photogrammetry (C); and subsequent 3D model in Photoscan (D). Orthophotos of the 3D model: (E) top view of whole fallen tree (gap on RHS was a section removed to measure trunk hollow, but it was included in the root:shoot ratio calculation); (F) top view of rotated model showing cross-section at 1.3 m (red outline) and convex hulls (visible and estimated total, brown lines); (G) side view of model showing root volume decrease with depth.

**Figure 6.**
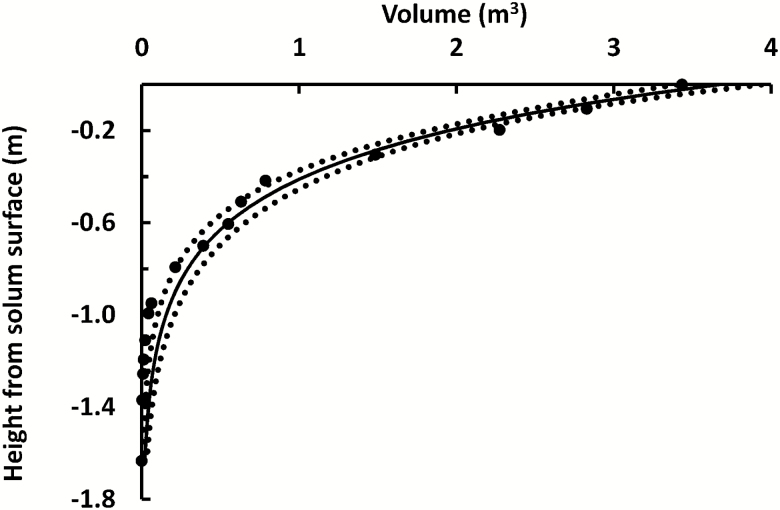
Geometric analysis from 3D model of myrtle tree in Fig. 5: ERV as a function of height from the mineral soil surface.

Three dimensional models of 29 large (mean DBH = 4.46 m, range 2.83–7.16 m), *in situ* eucalypts: (25 *E. regnans*, two *E. obliqua*, one *E. regnans*–*E. obliqua* hybrid, and one was either *E. regnans* or *E. obliqua*; unidentifiable due to logging and subsequent burning) were made using digital-SLR photography and Photoscan (Agisoft 2015, versions 1.0.4.1847 to 1.2.0.2198), and were analysed using Photoscan and ArcGIS (Figs 2–6).

Photoscan uses a self-calibrating photogrammetric bundle adjustment to solve for the camera geometry (camera calibration, including focal length and lens distortions) and to compute camera location and orientation data for each camera exposure station (Kraus 2007; Verhoeven 2011; Luhmann *et al.* 2016). It then uses advanced multi-station image matching techniques to derive a 3D dense point cloud, based on points seen on the surface visible in multiple photographs, from which it constructs a 3D mesh model of the object’s surface. No automatic or semi-automatic model segmentation is required.

Trees were selected so that the allometric equations would cover a range of tree sizes (including the high end) and to represent the prominent components of tree architecture, including response to ground slope, which averaged 8° (range 0–24°). There were 20 live trees and nine cut stumps from logging, 14 measurable hollows and 20 undisturbed humus mounds. Four live trees had large basal fissures through which one could walk (‘walk-in’ trees). Examples of eucalypt roots, loose logging debris, myrtle trees (*Nothofagus cunninghamii*) (nine), sassafras trees (*Atherosperma moschatum*) (one), fallen trees, branches, root and trunk slices, and soil profiles were also modelled in 3D. Only the eucalypt trees had humus mounds large enough to measure. Measurements in 2D, from earlier work (Dean 2003; Dean and Roxburgh 2006) of three eucalypt buttress ‘logs’ with hollows, in logging debris, were added to the data set.

### Photogrammetric procedure

Photographs for use in Photoscan can be acquired using a ‘normal’ geometry with camera (principal) axes approximately parallel for all camera locations, or with a convergent geometry with camera axes converging towards the object to be mapped (Kraus 2007). Convergent geometry provides a significantly stronger geometric solution, leading to a more accurate camera calibration and stronger ray intersections. A convergent camera geometry was adopted in most of the present work. It was combined with ‘normal’ geometry, in accordance with the Photoscan instructions (Agisoft 2015), in order to map the diversity of surfaces, such as fallen trees and the cavernous interiors of hollow trunks.

For each object to be modelled, multiple, overlapping photographs were acquired over the whole region of interest, taken mostly perpendicular to the different surface sections. For each eucalypt tree, between 143 and 846 photos (average 375, range 143–846) were acquired of the buttress regions and partial upper trunks with a digital-SLR camera, from ground level. The camera principally used was a 24.1 MP Nikon D3200 and AF-S Nikkor 18–200 mm lens with image stabilization. For one eucalypt tree a different camera was used: a Pentax 14.5 MP K20D and Pentax DA 18–55 mm lens. The Nikon camera had a Solmeta GPS (Geotagger Pro 2) attached for geotagging the file headers of photos, recording routes to trees and to aid in photo orientation if necessary. For photography for use in Photoscan the zoom lens was fixed at the shortest focal length (18 mm). The photography of large stumps and root balls required climbing over them, and taking photographs while ascending and descending in order to connect the models derived from different vantage points.

Prior to photography for 3D modelling, photogrammetric targets were placed around the object to be modelled, e.g. the buttress region of trees. These targets were of two types: (i) two scale bars placed on opposite sides of the object, each containing two targets separated by 1.200 m, that provided metric information used to control the scale of the photogrammetric model and which doubled as tie points, and (ii) additional tie points, the positions of which were not measured in the field but were well-defined, unambiguous targets observable in multiple photographs **[see****Supporting Information—Fig. S3****]** and which improved the strength of the photogrammetric solution. The second type of photogrammetric tie points positioned in the forest was mostly small, differently coloured plastic spheres of contrasting colours and size, from 3 to 15 mm diameter fixed to the surface using a nail **[see****Supporting Information—Fig. S3****]**. The colours were crucial to identification and differentiation on photographs, taken with either sky or vegetation in the background, and in different lighting. The visualized centre of the sphere was used as the tie point. A third set of tie points, not intentionally placed, being either natural features or features on field apparatus etc., were identified during image processing in Photoscan. The average total number of photogrammetric targets used per model, including those on the scale bars, was 15 (range 4–37).

The scale bars were light-weight, 1.5 m long, and each had two screws drilled through, 1.200(±1) m apart. The screws served three critical purposes: allowing the stake to grip the side of the tree, providing targets for the subsequent photogrammetry and acting as scale marks. Pink flagging tape was wrapped around the screw-bar join to allow contrast detection during image processing. A length of screw was left proud of the flagging tape so that the junction of the screw axis and surface of the bar (i.e. the target) could be visualized during image processing **[see****Supporting Information—Fig. S3****]**. During image processing these targets were located to the nearest ~0.5 mm, in the closer images. Some larger models required the placement of the scale bars in more than one location in the field, with subsequent merging of the corresponding submodels during image processing, using common tie points.

The photos were photogrammetrically aligned, most often starting from a good approximation achieved automatically by Photoscan, followed by accurate manual identification of each ground control point and tie point on each photograph (wherever they were visible). The latter process was iterative as the initial automated placement of new tie points improved as the number of correct camera positions increased.

Quality control of the accuracy and precision of the modelling were achieved by correcting photo alignment using as many tie points as necessary, to provide a result of only one realistic model fragment to which all photographs contributed. An indication of the quality of the solution was the derived length of each of the two scale bars. The average difference in length between the two scale bars measured in processed imagery was 2.3 mm (range 1.4–7.7 mm), i.e. a relative distance error or length distortion across the width of the 3D model of 0.19 % (range 0.011–0.64 %).

Each model improvement step in Photoscan consisted of photo alignment, dense point cloud generation and mesh generation, which typically took ~24 h. The maximum number of facets (indivisible planar surface elements) for final model construction was set at 5200000. Complete processing took ~1.5 weeks per model on a Windows PC, with 8x Intel Core i7-3820 3.60 GHz processors, 64 GB RAM and a NVIDIA Geforce GTX 670 graphics card.

Once the photos had been correctly aligned and maximum accuracy and precision achieved, models were oriented in Photoscan to their natural vertical orientation using a combination of thin, background trees (vertical) and the diameter tape (horizontal). Vertical translation was performed to place the average diameter tape location at 1.3 m above the top of the mineral soil which was given the height of 0 m (in accordance with the forestry standard used during fieldwork).

The model data were exported in 3D colour format (.wrl, .pdf and .obj), and 2D format for standard views: GeoTIFF (orthophoto with 2 mm object space resolution) and digital elevation model (DEM). The 3D formats were used in visualization to record or reveal ecological aspects, and the 2D formats were used for shape analysis in ArcGIS. The 3D models also provide a data archive for future use (such as stem taper and moss area) in case the trees are destroyed by logging or fire.

The orthophotos and DEMs were imported into ArcGIS. Geometrical attributes determined in the GIS were DBH (after correction for humus, large epiphytes, burls, coarse woody debris (CWD) and the diameter tape), cross-sectional area (enveloping area including bark), hollow area, footprint area, ground slope, and humus mound area and volume (Fig. 2). Allometric equations connecting DBH and ground slope to these other geometric attributes of tree architecture were derived using Eureqa (Schmidt and Lipson 2009; Cardoso *et al.* 2015). Specific equations were selected from a range of analytical models suggested by Eureqa. Selection was through compromise: those of higher coefficient of determination (*R*^2^) but still not too complex, i.e. as few parameters as possible and where gains by small decreases of *R*^2^ were no longer sufficient to warrant extra parameters. Parameters in the equations were refined by non-linear regression in LABFit (da Silva and da Silva 2015), which uses the Levenberg–Marquat algorithm for least-squares, optimizing the sum of squared errors in *y*.

Hollow area and cross-sectional area were determined as functions of DBH; footprint was determined as a function of DBH and slope; and humus dimensions were determined as a function of footprint. Double and floating point precisions were used in calculations throughout, so as to minimize accumulation of rounding errors. Values for equation parameters are given in the manuscript with more precision than justified by the standard deviation in order to allow readers to use the same values in their work without introduction of additional error due to rounding here.

### Retrieval of buttress-region data using GIS

It was necessary to correct the field-measured DBH due to possible incorrect placement of the tape measure around such large girths (up to 22.5 m). This was achieved by GIS analysis of the 3D models (using ArcGIS). Contours at 0.1 m intervals were created in the GIS, including one at the height of the DBH tape (1.3 m). That 1.3 m-level contour was corrected ArcGIS for humus on top of the bark, large epiphytic trees, burls and for the diameter tape itself, which the Photoscan solution sometimes included as part of the model, near buttress spurs. When the 1.3 m-level contour was thus adjusted a convex hull polygon was calculated that modelled the ideal placement of a DBH tape, its perimeter giving a more accurate measure of tree DBH than measured in the field. That corrected DBH was used in derivation of allometric equations. The DBH of one *E. regnans* could not be measured in the field due to proximity of a mature, epiphytic myrtle, with both trunks joined to ~5.4 m above ground. Instead the DBH was measured from a cross-section of the 3D model viewed in the GIS, with subtraction of the epiphyte by manually re-digitizing that section of the contour at 1.3 m **[see****Supporting Information—Fig. S4****]**. The measurements in the GIS allowed the area enclosed by bark at 1.3 m and the hollow area for stumps and walk-in trees to be determined. Data from three buttress logs in logging debris were included in the hollow calculation. Formulae for hollow area and bark-enclosed area were determined as functions of DBH.

Footprint and humus mound dimensions were also measured using GIS. An approximate perimeter of the footprint was indicated by where the contour lines no longer showed bumps due to the lateral roots. It was refined by on-screen digitizing. Ground slope was measured from the model’s DEM using the 3D coordinates of the highest and lowest points just outside of the footprint.

Humus volume was determined by locating the area of humus (as seen in vertical projection) in the flutes between spurs using the GIS (Fig. 2). The areas were delineated by digitizing the representative small polygons between the bark of the flute and the convex hull polygon at 1.3 m height (representing the ideal DBH tape placement). The slope of the surface of the humus mound was determined by drawing radiating lines from the bark pointing away from the centre of the tree to the forest floor, and overlying the centre of the humus filling the buttress flute. The lines were projected onto the humus surface using ArcGIS 3D Analyst. The vertical profiles of the lines were then exported to MS-Excel and corrected for any bumps due to debris such as fallen branches. Of the 29 eucalypts modelled, 20 were suitable for determination of humus volume. The humus mounds of the others eucalypts had been burned or otherwise damaged during or after logging.

The humus mounds of the different trees had a common slope, which was linear: height/length = −0.8(0.2), or ~38(9)°, (standard deviation in parentheses), *N* = 20, *R*^2^ = 0.98. The linearity and common slope simplified the determination of humus volume. Although the humus mound in a flute spreads sideways further from the trunk and meets humus from neighbouring flutes, its area outside of the convex hull (of the diameter tape) at 1.3 m height was approximated as a rectangle. The sides of the rectangle were parallel and perpendicular to the edge of the convex hull (Fig. 2). Another approximation made was that the flute area inside the convex hull could be represented as a triangle. The triangle had the same area, and base length along the convex hull, as the observed, irregularly shaped flute. The vertical sides of the humus in each flute were approximated as vertical. Thus, there were two humus volumes to calculate per flute: a triangular prism with a sloping top, and a rectangular prism with a sloping top. In some instances there was no humus in the flute (e.g. the large flute on the entry side of a walk-in tree) or the humus mound did not reach the convex hull (in which case there was only the triangular prism to estimate). The parallel-sides approximation of the rectangular prism part of flute-humus, which underestimated the amount of humus, was intended to compensate for the approximation of a vertically sided triangular part of the flute, which would generally overestimate the amount of humus, as the sides of the buttress roots often spread inwards below the surface of a deep humus collection.

### Subtraction of coarse root volume near trees

#### Generic tree species.

The below-ground root volumes in the buttress region of trees were estimated—the ‘effective root volume’ (ERV)—in order to adjust the typically measured soil bulk density and SOC per unit area for the soil displaced by those roots. That calculation required root volume to be formulated as a function of DBH and of depth. For that purpose a combination of the 3D modelling, tree packing geometry, data from the literature (Ashton 1975; Nicoll *et al.* 2006) and formulae from Dean *et al.* (2012b) were used.

As a first approximation, and for a default value in the absence of other evidence, a moderate rate of 2.861 m^−1^ for the exponential fall-off of root volume with depth (‘root fall-off rate’, RFR) was chosen from the literature. This value was for 40-year-old Sitka spruce on sloping terrain (Nicoll *et al.* 2006 their Fig. 5). Determination of the RFR under tree trunks requires excavation and consequently literature data for the phenomenon are sparse. This age of tree was considered appropriate because it has a smaller lateral root volume than a mature tree and thus represents more the central region under a mature tree, which is the region of interest in the present work. The data presented in Schwarz *et al.* (2010 their Fig. 4) fitted an exponential RFR of 4.4889 m^−1^ but were for the whole root systems of boreal and cool temperate forests, probably of various ages. A lower RFR has been recorded: ~1.5 m^−1^ (Eamus *et al.* 2002), probably due to a lower moisture-balance environment (Canadell *et al.* 1996). Limited literature data and the similarities mentioned above led us to believe that the Sitka spruce datum was the most suitable first approximation to employ.

A single RFR is an approximation for the combined RFRs of tap, sinker and lateral roots; and the rate below the buttress region is more dependent on taproot or sinker roots than it is for the root system as a whole. The SOC fall-off rate (SFR) used was from data for the Styx Valley (Dietrich 2012). The percent reduction in SOC per unit area was determined by dividing the soil and the root volume into fine depth increments, subtracting the soil volume corresponding to root volume, then recalculating the SOC per unit area, and comparing with the original SOC per unit area.

To determine the pathway for further development of the methodology, a sensitivity analysis of all parameters (fall-off rates, root mass and depth increment) was performed using stand-level root data for old-growth Douglas-fir (Santantonio *et al.* 1977). The stand-level data for old-growth Douglas-fir (Santantonio *et al.* 1977) were combined with the published SOC data for the Styx region (Dietrich 2012) and the RFR described above (Nicoll *et al.* 2006 their Fig. 5). Equations representing trends were found using Eureqa and the simplest solutions were chosen that had *R*^2^ = 1 within five significant digits. The sensitivity analysis showed four major trends that were helpful in further modelling and for stand-level calculations:

(1) The percentage SOC reduction at the stand level when accounting for root volume decreased with step size and changed in the third significant figure if vertical step size was >0.05 m (while keeping root volume constant). All further calculations were done with a 0.001 m step.

$$SOC\_reduction\%=4.5778-(1.0970step^{1.6506})$$ (1)

(2) The percentage change in SOC per hectare was positively correlated to the root-volume-falloff exponent (i.e. shallower-rooted trees decreased the SOC per unit area more than deeply rooted trees, for the same total root volume, which is logical as SOC density is higher nearer the surface).

SOC_reduction%=6.8656RFR/(1.4152+RFR) (2)

(3) The percentage change in SOC per hectare was linearly proportional to ERV per hectare.

SOC_reduction%=0.0074888ERV (3)

(4) The percentage change in SOC per hectare increases with the SFR with depth (while keeping root volume constant) but with an asymptote of ~13.7 %. It was approximately linear within the bounds of typical SFRs (i.e. 1–2). This simplified the calculation of different root volume effects for different SFRs.

SOC_reduction%=13.717−39.781/(2.9124+SFR) (4)

#### Eucalyptus roots.

Typical root architecture and volumes were estimated from Ashton (1975), from observations of the root architecture of numerous trees upturned during logging or by logging plus windthrow, and from 3D modelling of the buttress and roots of five logged trees using Photoscan. In summary, as the *E. regnans* tree matures the central tap root or tap roots decompose and are replaced by a separate sinker root adjoining each major lateral root, with these laterals forming the spurs of buttresses. Within the footprint there are also sometimes much smaller sinker roots further along each lateral.

From the present observations the width of *E. regnans* sinker roots was ~⅓ that of lateral roots. The ‘summary sinker’ is all sinkers added together for a given lateral **[see****Supporting Information—Fig. S6****]**. The lateral and sinker roots were modelled as cones, one summary sinker per lateral and 20 laterals per mature eucalypt tree. The choice of 20 laterals, each with one sinker root, followed observations of mature and senescing-mature *E. regnans* trees (Ashton 1975 and the present work), combined with approximations necessary for mathematical modelling. Horizontally, these laterals began at a distance of ~DBH/2 from the tree centre. The length of the laterals was held constant at 11.3 − (DBH/2) m, with the value of 11.3 obtained from Ashton (1975) (9–13.6 m). Additionally, 11.34 m is the radius of packing non-overlapping circles, each representing a *E. regnans* tree in a 400-year-old stand occupying 1 ha, assuming the stand density of 18.61 trees per hectare from formulae in CAR4D (Dean *et al.* 2003). It is also the radius of equivalent circles representing the non-overlapping, space-filling area occupied by a stand of 320-year-old trees of ~25 trees per hectare, i.e. close to the 27 trees per hectare for a densely packed stand >300-year old (Gilbert 1959). Inter-tree distances for *E. regnans* are of course not regularly spaced nor free of root overlap (Jarrett and Petrie 1929 and observation of juxtaposed trees in the present study), but it is expected that root overlap would be minimized where possible (Schwinning and Weiner 1998), as is canopy overlap, achieved during self-thinning (McMurtrie 1981; Enquist *et al.* 2009).

The laterals are concentrated at 0.3 m depth (Ashton 1975) and therefore the diameter of the lateral from which sinkers develop at the start of the lateral was set at 0.6 m (i.e. from the bottom of the lateral). The summary sinker roots were modelled as finishing at 2.55 m depth, from Ashton (1975), and from observation in the present work of ~2.5 m long sinker coarse roots on upturned trees. The RFR of *E. regnans* was calculated for this approximate model. These approximations contrast with the precision of the buttress-region photogrammetry but the high precision of that work carries through to the stand-level calculations and the equations are available for use in other studies.

The total root volume for *E. regnans* was determined from formulas in program CAR4D (Dean *et al.* 2003) as a function of age, and the comprehensive output of annual stand attributes from CAR4D was reformulated as a function of DBH using Eureqa:

$$Er\_total\_root\_volume=2.33260DBH^{2}$$ (5)

The affected region (where SOC is usually not measured near a tree) was assumed to have a radius 1.5 times that of a circle of equal area to each tree’s footprint. This value was chosen subjectively, based on observations and modelling of bared roots, and in the absence of any other information. The root volume within this region, i.e. the ERV (being part of the total root volume), was partitioned between the lateral and sinker roots, trimmed to the 1.5× footprint, split into 0.001 m vertical increments, and its effects summed over depth. The effect on SOC per unit area, for a range of DBHs (1–8 m), was reformulated using Eureqa for use at the stand level.

The *E. regnans*-dominated forest contains in places some *E. obliqua* and *E. delegatensis*. For these two species the same area around the trunk affecting SOC measurement as for *E. regnans* was assumed (1.5× footprint where footprint was calculated as for *E. regnans*). Their root volumes were calculated using formulae for above-ground biomass and assuming 15 % of total biomass was roots (Dean *et al.* 2012b), which was an average value 10.5–19 % (AUSLIG 1990; Carnahan 1977; Feller 1980; Mokany *et al.* 2006). Wood densities were from Ilic *et al.* (2000). Effective root volumes were determined as a function of DBH, assuming the same geometry as for *E. regnans*, and using Eureqa to determine suitable functions for stand-level use.

#### Rainforest species roots.

As a test of the assumed value for root:shoot ratio and RFR, the above-ground and below-ground volume of one whole, mature myrtle of DBH 1.76 m and height 32(±4) m, was measured. It was compared with a theoretically derived value from more general, generic data. Being only one tree, the process is also detailed here to demonstrate the usefulness of the methodology for future research. The trunk hollow was subtracted from the outer surface volume. It was a mature, senescent tree pushed over during logging. Its major lateral roots had snapped off at about the distance from the tree centre within which SOC is typically unmeasured—meaning that the observable roots constituted the ERV.

Soil was carefully removed from the roots with hand tools (Fig. 5). A 3D model of the root system and lower trunk was created from 356 photographs using Photoscan and reoriented to its original, upright position (Fig. 5). Roots greater than ~0.01 m diameter were captured in the model. The model was divided into 16 horizontal segments, ~0.1 m deep, and the root volume was measured for each segment in the 3D model using Photoscan. An exponential function for volume ERV as a function of depth was determined by non-linear regression (Fig. 6) and the ERV was 3.67 m^3^.

The convex hull area of soil enclosed by the roots, as seen from above, was determined using GIS. The root model was merged with a second model, being that of the whole above-ground portion of the tree. The branches had been broken but the leaves and fine twigs had dried and fallen and therefore it was possible to measure the whole trunk and branch volumes by importing the DEM and orthophotograph of the 3D model into the GIS. The volume of the whole tree was also calculated from the 3D model using the volume calculation algorithm in Photoscan. The two volumes were not significantly different and their average was used in further calculations.

To determine the root:shoot ratio for the whole myrtle, major lateral roots were modelled as 10 m long cones. That distance corresponded to the distance observed where major lateral roots of myrtles tapered to ~0.005 m in diameter. Total root volume was 5.5 m^3^, and ERV was therefore ~66.7 % of total root volume. The dimensions of the pushed-over myrtle were: above-ground volume 30(±4) m^3^, total volume 36(±10) m^3^, fraction roots 15(±4) %, root:shoot ratio 0.18(±0.05).

The average length of coarse roots (to their point of snapping) was 2.59(0.4) m (*N* = 42 points, standard deviation in parentheses), as measured from the tree centre. The area of a circle enveloping roots of that radius was 21.1(±3) m^2^. The area of the irregular shape projected by the roots, including an estimate of those not visible and still below ground, was 19.4 m^2^. These two values are estimates of the area influenced by the ERV.

The empirical derivation above for ERV was compared with a theoretical value derived from the above-ground biomass for rainforest species. The above-ground biomass was calculated using Equation (4) in Dean *et al.* (2012b), and the root biomass was assumed to be 15 % of total biomass (Dean *et al.* 2012b). A wood density of 577.3 kg m^−3^ (Ilic *et al.* 2000) was used to calculate root volume, and the ERV was calculated as 66.7 % of total root volume (as for the modelled myrtle tree). The estimated root volume for the pushed-over myrtle was then calculated to be 6.6 m^3^. This was considered to be close to the empirical value of 5.5 m^3^.

## Results

### Above-ground buttress region of eucalypts

The average correction between the DBH measured from the 3D models and the field-measured DBH was −0.25 % (−15 to 7.6 %, *N* = 28, using absolute values: average correction = 3 %, SD = 4 %). The data showed that there was a general but non-significant trend of underestimation of larger-tree DBH in the field and overestimation of the smaller trees. This suggests that for most trees, including the large ones, our method of DBH measurement in the field would not significantly overestimate carbon stock. The two largest corrections of −14 % and −15 % were due to burls and immovable logging debris, respectively. The DBH for these two trees could not have been so readily calculated without use of the 3D model.

Parameters for equations are given in Table 1.

**Table 1.** Parameters for equations derived from empirical data.

Equation	*a*	SD	Units	*p*(*t*)	*b*	SD	Units	*p*(*t*)	df	*R* ^2^	*P*
Equation (6)	1.93190	0.05	None	<0.0005					28	0.88	<0.005
Equation (7)	1.78703	0.06	None	<0.0005					28	0.85	<0.005
Equation (8)	1.10367	0.3	m	<0.0005	1.02874	0.006	None	<0.0005	19	0.76 adj.	<0.005
Equation (9)	2.33694	0.9	m^3^	0.02	0.0267006	0.006	m^−2^	<0.0005	18	0.52 adj.	<0.005
Equation (14)	3.67546	0.1	m^3^	<0.0005	3.16199	0.2	m^−1^	<0.0005	14	0.98 adj.	<0.005
Equation (16)	0.6337294	0.01	None	<0.0005					31	0.97 adj.	<0.0005
Equation (17)	1.20573	0.5	m	0.043	−3.16758	2	m^2^	0.206	12	0.24 adj.	0.04
Equation (18)	0.284597	0.03	None	<0.0005	3.59490	0.8	None	0.001	19	0.74 adj.	0.0002

**Figure 7.**
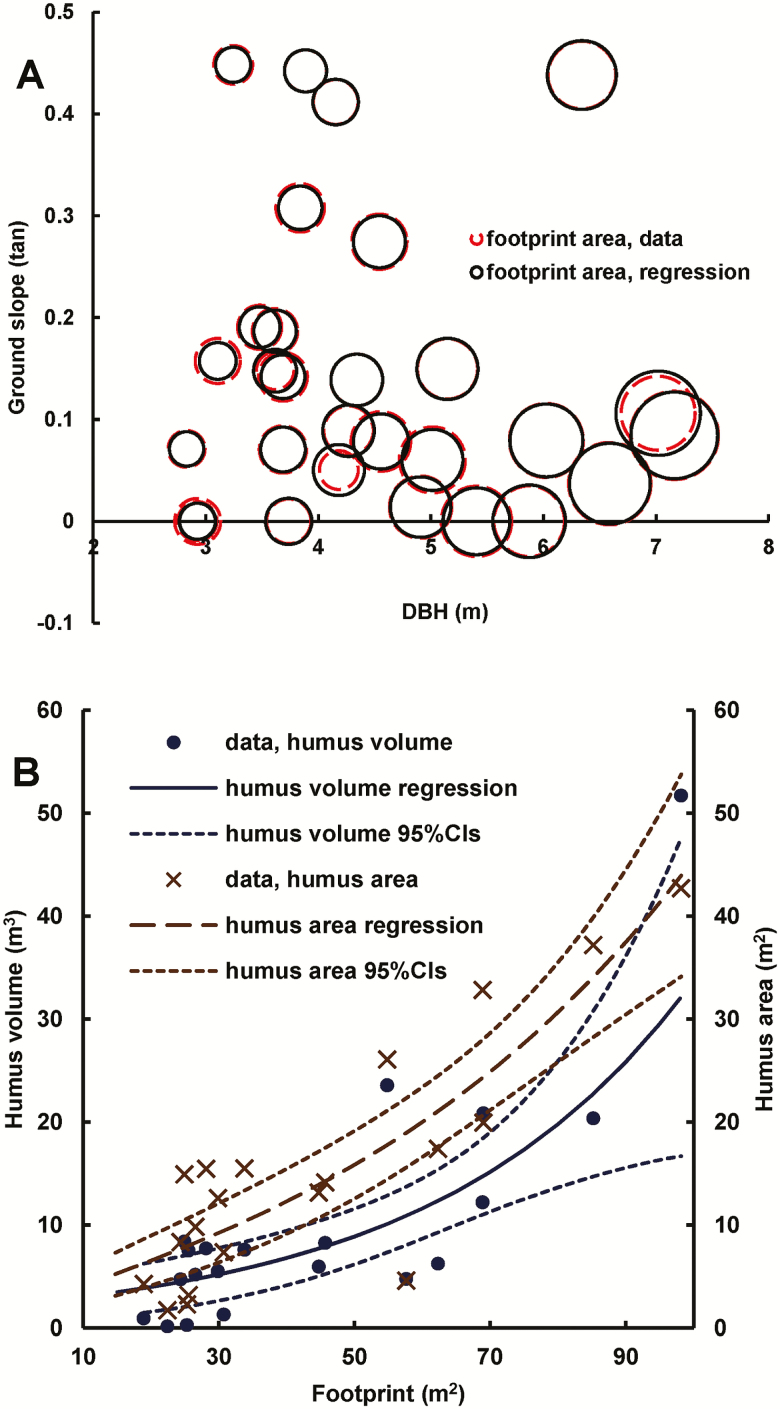
Some attributes correlated with individual tree measurements. (A) Footprint of eucalypt trees (represented by circle size) versus DBH and ground slope. The 95 % confidence intervals, corresponding to 2.6 % of the one parameter, were too narrow to show at this scale. (B) Humus area within the humus mound of eucalypt trees and the corresponding humus volume, as functions of footprint.

The footprint of eucalypt trees was given by (Fig. 7A):

$$footprint=DBH^{2}(a-slope)$$ (6)

where footprint is in m^2^, DBH is in m, and slope is tan of the ground slope angle. The negative correlation with slope concurs with Nicoll *et al.* (2006) where downhill lateral roots descend more quickly into the soil on steeper ground.

For reference purposes, if slope is not measured, and Equation (6) cannot be used, then:

$$footprint=aDBH^{2}$$ (7)

where footprint is in m^2^, DBH is in m.

The humus area within the humus mound, which envelopes the buttress region, and the humus volume above that area were both functions of the footprint (Fig. 7B). Humus area within the humus mound was given by:

humus_area=a(footprint bfootprint)12 (8)

where humus_area is in m^2^, and footprint is in m^2^ given by Equation (6). Humus volume, above humus_area, was given by:

humus_volume=a exp(b footprint) (9)

where humus_volume is in m^3^ and footprint is in m^2^ given by Equation (6). Large buttress roots occupied 49(15) % (standard deviation in parentheses) of the whole footprint, and humus area covered 40(19) % of the area between the projected stem basal area and the footprint perimeter.

The equation for SOC in an individual tree’s humus mound is:

humus_SOC=a humus_volume (10)

where humus_SOC is in kg and humus_volume is in m^3^ as given by Equation (9), and *a* is the humus mound SOC density in kg m^−3^.

### Effective root volume

Effective root volume was a species-dependent function of DBH. The RFR for the eucalypt model with conic frustum laterals and conic sinkers was 3.133 m^−1^, and that for the single myrtle empirical examination was 3.182 m^−1^. These were considered similar (within experimental error) to the value of 2.861 m^−1^ from Nicoll *et al.* (2006). For eucalypt roots the modelled value could be used, but as only one myrtle was examined experimentally there was insufficient reason not use the value of 2.861 m^−1^ for non-eucalypt species. The linear proportionality between ERV per hectare and effect on SOC per hectare (Equation (3)) simplifies stand-level calculations in the tallying of root volume effects for individual forest plots. With the slightly higher (RFR) of 3.133 m^−1^ for eucalypts, the parameter in Equation (3) increases to 0.0098756. For eucalypts in a stand, Equations (11–13) (*E. regnans*, *E. delegatensis* and *E. obliqua*, respectively) and Equation (3) can be used to tally the total ERV per hectare and the corresponding percentage reduction in SOC due to the roots below the buttress region:

$$Er\_ERV=\;0.936544DBH^{1.29339}+299.187\exp(-12.6115/DBH)$$ (11)

Ed_ERV=40.0675DBH/exp(7.23774/DBH) (12)

$$Eo\_ERV=1.82528DBH^{2.33589}$$ (13)

These equations appear a little complex compared with those for humus and footprint but the ERV is a subset of the entire root volume, with its measurement boundary chosen for pragmatic reasons. For the example myrtle tree, from modelling the ERV as a function of depth:

$$understorey\_ERV=ae^{-bz}$$ (14)

where understorey_ERV is volume in m^3^ and *z* is the distance from the mineral soil surface in m (parameter values in Table 1). For understorey trees with DBH ≥ 1 m, when doing stand-level calculations, ERV can be calculated assuming linear proportionality with DBH^2^ to that for the modelled tree (as in Equation (5) for *E. regnans*):

$$understorey\_ERV=3.67097{\left(DBH/1.76\right)}^{2}$$ (15)

When using Equation (15) for understorey trees with DBH < 1 m in a typical forest stand the root volume appeared to be overestimated compared with expected values for that biome from the literature and therefore the eucalypt formulae Equation (11) should be used for understorey trees (with DBH < 1 m).

### Cross-section

**Figure 8.**
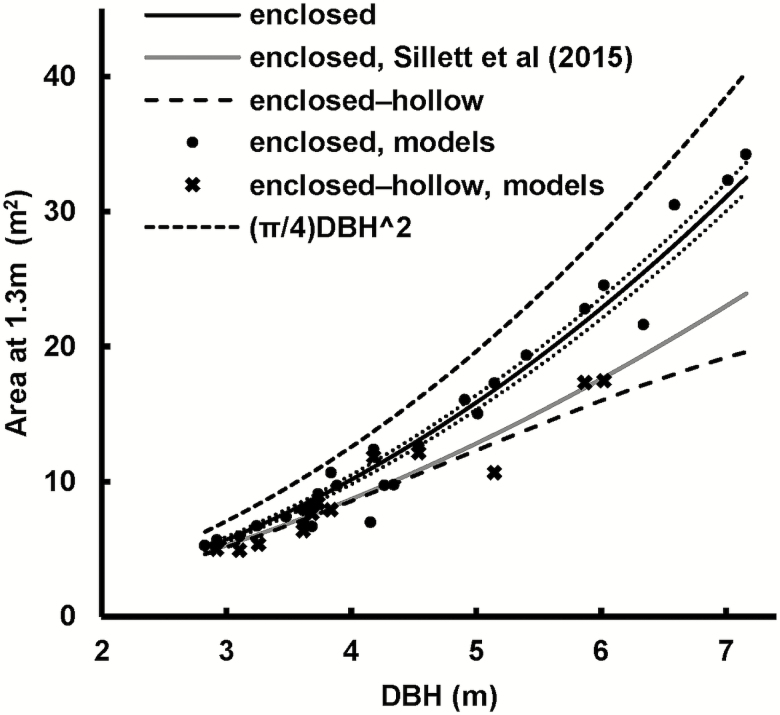
Potential wood area at 1.3 m versus DBH. Solid, black line is regression fit to 3D models, dotted lines are 95 % confidence intervals. ‘enclosed-hollow’ = enclosed minus hollow. Gap between upper black dashed line and black solid line = acircular area deficit.

The deviation of the enclosed area from the area of a circle with the same DBH, due to flutes in the buttress and general acircular shape of the stem (the ‘acircular area deficit’ at 1.3 m), is the gap between the solid line and lower dashed line in Fig. 8.

The potential enclosed cross-sectional area at 1.3 m rose linearly with DBH^2^, though not as steeply as for trees with circular cross-section, due to the acircular area deficit (Fig. 8):

$$wood\_area=aDBH^{2}$$ (16)

where wood_area is in m^2^ and DBH is in m. (For circular trees *a* = *π*/4 ≈ 0.7854.)

**Figure 9.**
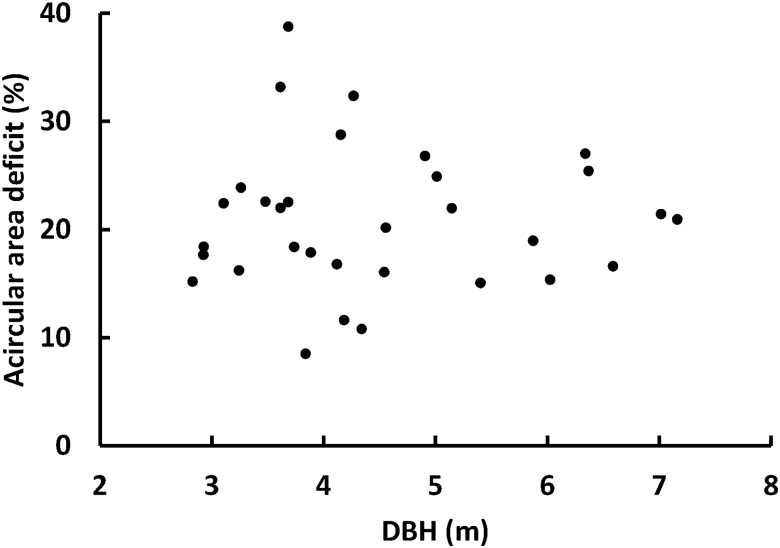
Acircular area deficit versus DBH. The deficit is likely to be more variable for younger and/or smaller mature trees. This variability will influence that of stand-level humus volume.

The deficit, as a percentage, was twice as variable for trees 2.8 ≤ DBH ≤ 4.5 m—21(8) % than for larger trees—21(4) % for trees with 4.5 ≤ DBH ≤ 7.16 m (Fig. 9). Saplings have a deficit closer to 0 %.

Hollow area at 1.3 m was given by:

hollow_area=(aDBH)+b (17)

where hollow_area is ≥0 and in m^2^, and DBH is in m. Without the two trees that were solid at 1.3 m (no basal hollow) the hollow area was given by:

$$hollow\_area={\left(aDBH\right)}^{b}$$ (18)

where hollow_area is ≥0 and in m^2^, and DBH is in m.

## Discussion

The dense point cloud and subsequent 3D surface provided by Photoscan ensured that the models were of sufficiently high spatial and colour resolution to yield serviceable tree allometry. High resolution was theoretically obtainable with multi-scan terrestrial LiDAR (MS) but the portability and access issues (Methods section) prevented its use. The need for multiple vantage points for the trees in this study when using MS concurs with work on buttressed trees in tropical forests (Bauwens *et al.* 2014; Bauwens *et al.* 2017). Handheld mobile scanning lasers (Bosse *et al.* 2012; Hosoi *et al.* 2013) are suitable for moderately difficult terrain and intermediate understorey densities but the measurement resolution is in the order of 0.02 m for small trees without epiphytes (Bauwens *et al.* 2014), which is too imprecise for deriving tree allometry, and it is likely to worsen in more complex environments. Additionally, the output does not provide high resolution colour information for visual identification of surface materials (e.g. to distinguish moss covering from the trunk). Mobile LiDAR technology may however be the most suitable for topography and stand-density mapping in support of aerial photography or airborne LiDAR over dense canopies. This surface detail and the portability of the equipment (i.e. camera, light-weight scale bars and small markers) make the photographic methodology described here superior to that available with terrestrial LiDAR (including handheld mobile), for forests with rugged terrain large trees, epiphytes, often dense understorey and a minimal crew.

Although our purposes were served by the photographic method in the present study, LiDAR is also useful in these forest types. We collected MS data for medium-sized trees (DBH 1 to 4.2 m) on the edge of logged areas, to enable derivation of detailed trunk and branch allometry: results will be reported elsewhere. HMLS data were collected in the same study area as the present study, to enable stand-level demographic interpretation, and that also will be reported elsewhere. The water displacement method used for checking LiDAR of root boles (Gärtner *et al.* 2009; Smith *et al.* 2014) was physically impractical for the large tree sizes in the present study (root lengths of ~11 m), and because the roots of large eucalypts were curled around neighbouring trees (Fig. 3).

The 3D models derived here also provide an archive for biological observations in the future (such as stem taper and moss area) in case the trend of demise of remnant large trees continues.

The methodology shown here sidesteps some destructive sampling. Ideally for volume calculation of the humus mound and lower trunk, a 3D model of a freshly logged tree would be made with the humus present, then the humus removed to reveal only the timber and mineral soil, and a second 3D model made. The difference between the two models would equate to the humus volume plus any buried CWD. This would provide more accurate allometric equations and therefore carbon accounting but would initiate carbon emissions and would kill the epiphytes.

MS and airborne LiDAR have been used to measure above-ground woody biomass (e.g. Kaasalainen *et al.* 2014; Calders *et al.* 2015; Hackenberg *et al.* 2015). Those studies validated their estimates using either destructive sampling or existing allometric equations. Although further destructive sampling would be unnecessary if using comparable LiDAR technology and data processing for the same forest types, it would need to be revalidated if working in very different forests. Root systems have also been studied with MS, but for trees much smaller than in our study. The diameters of trees and basal area of stands in the present study were on average an order of magnitude larger than those examined in other studies, where MS was used (e.g. Liski *et al.* 2014; Smith *et al.* 2014; Calders *et al.* 2015; Hackenberg *et al.* 2015; Bauwens *et al.* 2016). Additionally, the trees in the present study had a quite different physical shape and surrounding ecosystem architecture, e.g. fluted buttresses; large hemi-epiphytes; trunk and branch hollows; closed understorey; and broken, regenerating crowns. These attributes required a different experimental approach. However, MS may well be applicable to the upper parts (mid-stem and crown) of such trees if applied at the edge of fresh clearfells (prior to wind damage of crowns). The use of drones (UAVs) on such trees may also extend the method introduced here to their upper parts.

Some form of validation will always be required, if either the trees of a particular species are larger than those studied previously or if the mature trees experience a different fire regime. Where the intention is to provide a procedure for use in a rapid stand-level assessment (e.g. of biomass in a primary forest) then allometric equations will be required. The present study included examination of some trees felled or uprooted earlier during logging, so in effect the destruction part of the sampling was pre-performed.

Many allometric equations derived non-destructively miss the trunk hollow area (e.g. Sillett *et al.* 2015) and the non-circularity of the trunk perimeter. Knowledge of these attributes allows users of allometric equations derived from non-destructive sampling to have results as accurate as if deploying destructive sampling, but without the carbon emissions and conservation debt. The methodology used here allows calculation of such attributes, though for some hollow-area assessments trees were fortuitously found after logging.

**Figure 10.**
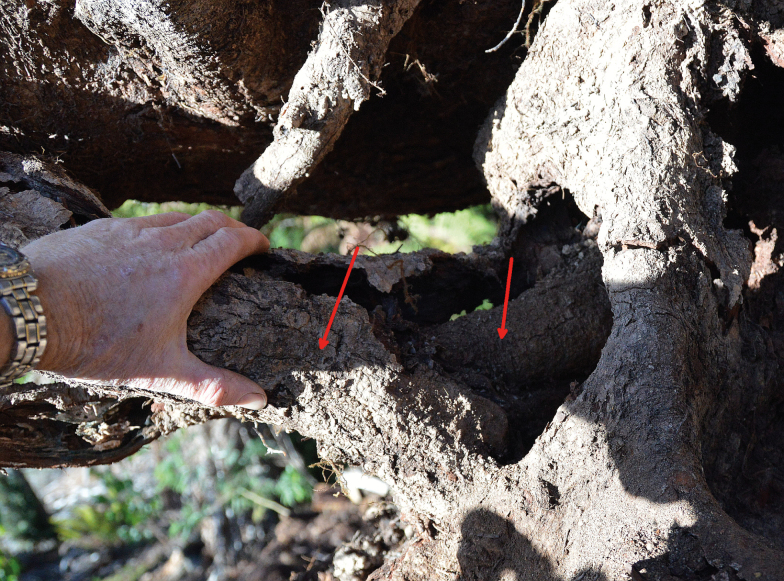
Roots growing inside larger roots reduce ERV. The displaced SOC by the roots will be less, but this is taken into account in the modelling presented here.

The formulae deduced here, for footprint, humus area, humus volume and area deficit, can be used at the stand level on inventory data, and when linked with example SOC data for such stands, can be used to provide adjustment to more-routine soil sampling of stands. In typical SOC assessments, SOC is sampled away from large trees and their main coarse roots. The roots encountered in those samples are used to adjust bulk density to give a SOC stock per unit area. The method assumes a uniform root density in between trees that also applies beneath tree trunks and buttresses. Root density in the soil is rarely published and consequently it is necessary to provide an adjustment for the soil bulk density (and SOC) based only on the ERV calculated in the present study. Thus, in effect, during calculations using formulae from the present work, the root volume under the trunk and nearby is duplicated by an amount equal to that typically found in between trees. Therefore, the method suggested here is conservative for estimating SOC per unit area. The roots of myrtles are more likely to occupy existing hollow roots than are those of eucalypts (as the exterior of myrtle roots appears to be more decay-resistant than the interior (Fig. 10), which was not observed for *E. regnans*)—this reuse of volume also reduces the soil displaced by roots and therefore diminishes the unaccounted-for SOC for a given DBH (though this was part of the present modelling). Some tropical trees exhibit similar growth patterns (Hallé *et al.* 1978). The SOC stocks adjusted for the forest attributes as shown here can be used to provide more accurate carbon accounts for primary forests and those subject to LUC.

The difference between Equation (17) and Equation (18) represents a substantial difference in forecast hollow area by including solid trees, though solid trees were exceptions (e.g. Helms 1945) and those solid trees measured in the present work were found over 50 years ago. With the increased prevalence of fire and logging since then, hollow-free trees are now less likely to be present. The historical situation is however important for modelling the effects of past emissions on current atmospheric CO_2_ concentration. It could be that such solid trees were too exceptional to include in a small sample size, and that a specifically targeted data collection during logging of a primary forest is warranted.

Errors can arise in measuring annual growth of mature *E. regnans* trees if using girth as an indicator because observations in the present work showed that they can grow new sapwood inside trunk hollows. It was noticed that the new, internal sapwood (and heartwood) grows near the flute folds, and it subtracts from the hollow area. New heartwood within the tree hollow in *E. regnans* was noticed up to at least 15.4 m above ground. Similarly, myrtles can grow inside their lower trunks. When this is combined with the fluted shape, only a portion of the radial growth will register on a diameter tape.

If applying the formulas derived here to other studies, where trees are measured only by their DBH, then the actual area of timber at 1.3 m height is given by Equation (16) minus Equation (17) (or Equation (16) minus Equation (18) depending on the prevalence of solid trees). It must be noted that the basal hollows generally did not go all the way up the trunk but were roughly conical in shape and followed the silhouette of the buttress taper. This means that when calculating tree biomass it should not be assumed that the cross-sectional hollow area at 1.3 m height is applicable to the whole trunk.

Our results differ from those of Sillett *et al.* (2015), in that the cross-sectional area deficit in the present work was smaller—our trees had more wood (Fig. 8). Their trees were smaller (maximum DBH of 5.53 m compared with our 7.16 m), younger and were mostly from Victoria (Australia). A higher area deficit can be an indication that the crown is more exposed to wind stress or the footing is unstable (Julin *et al.* 1998). The study by Sillett *et al.* (2015) focussed on tall trees, which may be subject to greater wind stress. Jaskierniak *et al.* (2014) showed a slightly larger area deficit of 27 % for Victorian *E. regnans* up to DBH of ~1.4 m, compared with the 21 % in the present work, and they concluded that the measured diameter is more erroneous as tree size increases. Similarly, the formulas in Sillett *et al.* (2015) imply that the percentage deficit increases with DBH. Conversely, Dean and Roxburgh (2006) suggested that the deficit reached 40 % for DBH > 3.34 m then decreased with DBH. Both Dean and Roxburgh (2006) and Sillett *et al.* (2015) used tapes to measure flute area, and therefore relied on fewer data and more interpolation. Results in the present work (Fig. 9) showed that the area deficit approaches a constant value for larger trees of 20.9(6.6) % and was more variable for smaller DBHs. Our data had a minimum DBH of 2.83 m and therefore did not reveal much of the increase for immature trees as did that of Jaskierniak *et al.* (2014). Combining the present study with that of Jaskierniak *et al.* (2014) suggests that the area deficit levels off between 1.4 and 2.83 m. In retrospect, the high end of the data of Jaskierniak *et al.* (2014) does show a slight trend for reduced deficit.

The higher variability in deficit for the low to mid-sized trees in the present work (Fig. 9) is likely to include data from some meta-stable trees that will die during further stand self-thinning. For larger trees, the reduced variation of deficit and convergence on the mid-range (21 %) suggests a change in the influence of wind on trees during stand development. The more-wind-stressed trees (those with the more prominent buttress flutes) have either died or had crown loss (with reduced wind impact thereafter), or the less-stressed trees (with <21 % deficit) have become the dominants and thereby endured an increase in wind impact. For more mature trees, without preferential spur development, equal growth of all external cambium means that the sides of the flutes grow towards each other. Thus, the sides of neighbouring spurs grow towards each other and occupy previous flute area, thereby decreasing percentage deficit area.

## Conclusions

Photogrammetric 3D modelling provided useful information on the buttress area and below-ground tree volume, which are amongst the most undocumented areas of mature trees, with regards to soil carbon assessment. It also provided further detail on the cross-sectional area applicable to tree carbon allometry. This new information on the physical attributes of mature trees means that the carbon forecasting models, such as CAR4D, which provide estimates of pre- and post-logging carbon stocks, can be updated. The 3D models developed here are irreplaceable, being for trees of a size that is becoming rarer and with the models being so detailed. The models could be useful to other scientific endeavours.

## Sources of Funding

The research was supported by the University of Tasmania, including by a Natural and Environmental Studies Elite Research Scholarship.

## Contributions by the Authors

C.D. designed experiments, performed analyses and wrote the paper; J.B.K. and J.O. assisted with project commencement and writing of the paper; all authors contributed to technical and/or fieldwork support and discussions.

## Conflict of Interest

None declared.

## Supporting Information

The following additional information is available in the online version of this article—

One file containing: Supporting Introduction (with photographs) describing forest ecology and land usage relevant to the tree anatomy and carbon accounting needs, Supporting Methods showing pictorially the finer detail of fieldwork in photogrammetry, and corresponding cited publications in Supporting References.

**Table S1.** Maximum diameter at breast height (DBH) used for calibrating publicly accessible, species-specific carbon allometric equations, compared with maximum recorded tree diameters for some common tall open-forest (TOF) canopy species. DBH is indicative of gross sequestered carbon over lifetime, and of flutes in buttress. Allometric equations are generally available only for trees up to around half of maximum size.

**Figure S1.** Legacy carbon from the earlier mixed forest. A *Eucalyptus regnans* log spans a creek centred in a 200 m wide gully mapped as rainforest—typical of the blurred spatio-temporal boundary between TOF and rainforest as seen from a carbon dynamics perspective. Cliff Creek, Styx Valley, Tasmania.

**Figure S2.** (A–C) Macroscopic above-ground mature *Eucalyptus regnans* tree architecture. Acute angles, averaging near 45°, where large branches meet trunks. Epicormic shoots have become large branches, forming lower crowns following stand self-thinning, crown volume has increased ((A) Styx and (B) Florentine Valleys, both trees were extirpated by logging—cannot be remeasured). Tree ‘(b)’ was ‘El Grande’ diameter at breast height (DBH) = 6.38 m, height = 75.4 m, photographed during logging. (C) Large myrtle epiphyte on right-hand side, trees on edge of logging coupe SX009C. (D) Sapling sassafras as hemi-epiphyte on *E. regnans*, joint to 3.9 m height above soil A horizon, Styx Valley. Tree is ‘Chapel Tree’, DBH = 6.03, height = 80.1 m. (E) Most epiphytes cut away, person stood on epiphyte roots, prior to logging, DBH = 4.95 m, in logging coupe SX004C.

**Figure S3.** Example ground control points (GCPs) and tie points used. Diameter at breast height (DBH) = 3.11 m. (A) Placement of GCP at the intersection of screw and rod at top of scale bar, and tie point on end of rod. (B) Tie points on distant and near objects. (C) Completed 3D model with large number of tie points needed for object’s complexity, variety of backgrounds (in photos) and range of ground elevation from top to bottom.

**Figure S4.** Wider use of 3D models. Model of *Eucalyptus regnans* diameter at breast height (DBH) = 4.56 m (coupe SX009C), in Supporting Information—Fig. S2C. The DBH could not be measured in the field due to the large hemi-epiphytic myrtle (left hand side in (A), right hand side in (B)). The myrtle could be separated in ArcGIS using cross-sections and contour levels, and the DBH then estimated, as shown in (B) (green line = 1.3 m contour, red line = corrected 1.3 m contour, blue line = 1.3 m convex hull, brown line = footprint). The lower, oblique view of the model is as viewed from uphill in the top view.

**Figure S5.** (A) Orthophoto created using terrestrial photography and Photoscan of a *Eucalyptus regnans* (diameter at breast height (DBH) 4.38 m, Tyenna Valley, coupe TN050E) root slice of large lateral within the footprint, ring age count = 350(±40) years: most expansive growth on top side, corresponding to buttress width expansion. (B) Google Earth® satellite image shows felled trunk, stump and neighbouring stumps, during logging (scale bar = 40 m).

**Figure S6.** Sinker roots on mature *Eucalyptus regnans*. (A) Large sinker root beneath edge of buttress and adjoining the large lateral (right-hand side of photograph). The area around the tree had been logged (coupe SX009C) and the tree had fallen and its buttress split open. (B) The tree in coupe TN050E had been logged. Soil from the coarse roots on one buttress spur was removed. Small sinker roots, not part of the buttress, were within the footprint zone.

Supplementary Material
