# Supplementary material for: Novel 3D geometry and models of the lower regions of large trees for use in carbon accounting of primary forests
Source: AoB Plants. 2018 Feb 28;10(2):ply015. doi: 10.1093/aobpla/ply015 (PMC5861447; doi:10.1093/aobpla/ply015)
Supplement: Supplementary Material [file ply015_suppl_supplementary_material.docx]

RESEARCH ARTICLE

**Novel 3D-geometry and models of the lower regions of large trees for use in carbon accounting of primary forests**

# SUPPORTING INFORMATION

# SUPPORTING INTRODUCTION

# Mixed-forest in Tasmania

The study area of the present work is in eucalypt tall open-forests with a rainforest understory (i.e. mixed-forest) in the maritime-temperate climate of Tasmania, Australia. Mixed-forest is common in the Australian State of Tasmania and was previously common in the State of Victoria, where it is now rare and termed ‘ecotone’ forest (Fedrigo *et al.* 2014; Petrie *et al.* 1929). Mixed-forest is a form of rainforest (Kirkpatrick and DellaSala 2011), though it is an overlap of tall open-forest (TOF) and traditional rainforest categories. The present work focuses on mixed-forests dominated by *Eucalyptus regnans*. The diameter at breast height (DBH, at 1.3 m) of *Eucalyptus regnans* (swamp gum/mountain ash) may reach ~7 metres in Tasmania, and in Victoria the historical maximum DBH was 10.8 m (Ashton 1975). However, the older stands in Victoria containing such trees have been replaced by livestock farms (Ashton 1975; Beilin 2007; Mainville 2007). *Eucalyptus regnans*-dominated forest is amongst the most carbon (C)-dense primary-forest worldwide (Fedrigo *et al.* 2014; Keith *et al.* 2009; Wood *et al.* 2010), mature individuals of *E. regnans* have the highest recorded, gross annual C sequestration rate globally (Sillett *et al.* 2015), and *E. regnans* is the tallest living angiosperm (up to 115 m Ashton (1975); Ashton (1981); Ferguson (1948); Mace (1996), being of similar maximum height to the tallest gymnosperm *Sequoia sempervirens* Sillett *et al.* (2010)). This makes *E. regnans* exceptional and these characteristics may also increase the signal-to-noise ratio when studying the influence of trees on soil organic carbon. Plus *E. regnans* has been targeted by industry (Dean *et al.* 2012) and therefore constitutes a major component of related carbon dynamics— two reasons for studying that forest type. Accounting of forestry’s industrial carbon emissions is important not only to determine the carbon fluxes accompanying present and future industrial activity but also in climate change modelling.

The area of tall open-forest (TOF) in Australia has decreased on net since Europeans first settled here (in ~1750) due to timber extraction, clearing for agriculture (Kirkpatrick 1986; Kirkpatrick 1994), and clearing for mineral mining and urbanisation (reducing temperate forest from 113 Mha to 55 Mha). Owing mainly to timber harvesting the remnant south-eastern Australian temperate forests are at 60% of their potential carbon stock (Roxburgh *et al.* 2006). Logging of primary *E. regnans*-dominated forests has been extensive in the States to which they are native, Victoria and Tasmania (Dean *et al.* 2012). Tasmania’s tall open-forests (TOFs), especially the more-mature *E. regnans* mixed-forests have been prized for pulpwood and lumber, initially for newspaper and then as the major source feeding the hardwood pulpwood market of the Pacific rim, especially Japan, until 2012 (ANM 1979; The Mercury 1941; WRI 2010; WRI 2014). Detailed climate modelling has confirmed an increased fire danger index forecast for Tasmania this century, especially for the region containing the most carbon-dense TOFs (Fox-Hughes *et al.* 2014). Reduced growth is forecast for these forests due to reduced water availability under climate change (Bowman *et al.* 2014). The increase in fire and decrease in growth both constitute net carbon emissions over time.

**Spatio-temporal difficulties in carbon stock assessment in Tasmanian tall open-forests**

Swamp gum (*Eucalyptus regnans* F. Muell) is still a dominant ecological component at over 500 years of age in mixed-forest in Tasmania (Wood *et al.* 2010), but without sufficient fire to initiate germination and sapling-stage eucalypts in place of the rainforest understorey (Ashton 1981; Cremer 1960; Gilbert 1959) the mixed-forest eventually becomes rainforest. Similarly, in the absence of stand-replacing fire, wet-sclerophyll can lead to mixed-forests (Ashton and Attiwill 1994; Gilbert 1959). With particular climate and fire frequency, a rainforest understorey of myrtle trees (*Nothofagus cunninghamii* (Hook. f.) Oerst.) in *E. regnans* mixed-forest can be replaced by young *E. regnans*, thereby increasing the carbon stock once the younger eucalypts reach canopy height (Mackey *et al.* 2008). However that combination may be more metastable with respect to fire and time than the mixed-forest, possibly requiring more-frequent low intensity fire for maintenance. For example Fedrigo *et al.* (2014) found marginally higher carbon stocks in mixed-forest than in wet-sclerophyll forests in the Victorian Central Highlands (VCH). Mixed-forests are often also uneven-aged with respect to the eucalypts (due to non-stand-replacing fire) (ANM c1960; Bowman and Kirkpatrick 1984; Turner *et al.* 2009), though even-aged stands of *E. regnans* mixed-forest are not uncommon (ANM c1960). It must be noted that the carbon allometrics (allometric equations) established for a low-fire-frequency location may introduce additional errors if used in a high-fire-frequency location (and vice-versa), due to tree-hollow enlargement with more-frequent or more-intense fires.

The documented area of rainforest has changed with the definition of rainforest, according to the percentage crown cover of eucalypt remaining within the mixed-forest (Hickey *et al.* 1993). In addition to the definition-dependent, blurred spatial boundary between the two there is also a blurred temporal boundary, as coarse woody debris and soil carbon, representative of either forest type, persists to a degree depending on its half-life. This material constitutes ‘legacy carbon’ (Harmon 2001; Harmon 2009) (Supporting Figure S1). Empirical studies attempting to compare soil carbon stocks in rainforest and mixed-forest will thus be futile (e.g. Dietrich 2012) until the transition between the two is almost complete for all forest attributes.


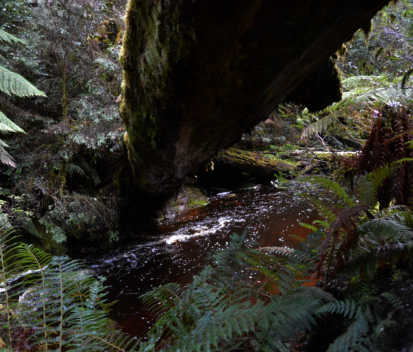


Supporting Figure S1 Legacy carbon from the earlier mixed-forest. A *Eucalyptus regnans* log spans a creek centred in a 200 m wide gully mapped as rainforest— typical of the blurred spatio-temporal boundary between TOF and rainforest as seen from a carbon dynamics perspective. Cliff Creek, Styx Valley, Tasmania.

Carbon stocks representative of any particular land-use and forest-type are best measured as a long-term, temporal average (IPCC 2003; Nabuurs and Schelhaas 2002). For TOFs the stand-level biomass oscillates in the long-term with natural wildfire (Dean *et al.* 2003). A considerable portion of killed biomass remains on site as coarse woody debris (CWD) and is not emitted to the atmosphere. While new biomass increases after fire and logging, the CWD decomposes sooner than if it had remained alive, except possibly for the small portion left as charcoal which may turnover on a centennial time scale (Singh *et al.* 2012), though charcoal is not long-lived in some microenvironments (Zimmermann *et al.* 2012). When assessing carbon stocks and their change, it can be difficult to differentiate between site index effects, logging history and stand age effects. For example in the Tasmanian timber production estate, TOF ≥110 years of age have on average 186 Mg ha^-1^ of carbon in aboveground biomass, and a maximum of 748 Mg ha^-1^ (Moroni *et al.* 2010). The interacting effects of fire and logging influence the landscape mosaic of such forests (Lindenmayer *et al.* 2009), with spatio-temporal implications for determining C dynamics. Carbon content varies spatio-temporally within one forest type, linked in part to how sporadic is fire, so spatial averages from small study areas are more likely to be different, for example for *E. regnans* in the VCH: 2.52 ha with 689 Mg ha^-1^ (Fedrigo *et al.* 2014), 0.729 ha with 706 Mg ha^-1^ (Sillett *et al.* 2015) and 3.18 ha yielding 1053 Mg ha^-1^ (Keith *et al.* 2009). Temporal averages are harder to empirically measure than spatial averages, due to relatively short human longevity compared with that of mature trees in primary forests. Comparison of rainforest and TOF total carbon stocks is problematic for this reason.

**The need for improved allometric equations for tall open-forests**

Over the last two decades carbon accounting of deforestation and industrial forestry has prompted research into allometric equations of large trees in relation to climate change modelling and carbon credits (e.g. Brown 1997; Chave *et al.* 2005; Dean 2003; Dean and Roxburgh 2006; Ngomanda *et al.* 2012; Nogueira *et al.* 2006). Missing information necessary for allometric equations suitable for carbon accounting for Australian timbers (Table S1) illustrates the paucity of scientific research into mature trees, with attention historically focused on young trees of the secondary forests, where financial investment is higher. Modelling carbon dynamics relevant to climate modelling requires parameterization of allometric equations for the full range of tree sizes and major (in terms of biomass) forest components. Accompanying forest usage and clearance, the size of the largest trees remaining in existence has been decreasing (Herrmann 2006; Lindenmayer *et al.* 2012). This makes the information gap smaller for current forests, but makes accounting for the earlier primary forests and our effect on them, and accounting for potential future forests, more difficult. The missing allometric equations means that effects of earlier forest usage cannot accurately be integrated into climate change modelling. The reader is referred to Melson *et al.* (2011) and Henry *et al.* (2015) for a comparison of error margins between different types of allometric equations and recommendations for improvement in allometric equations suitable for carbon accounting globally. In the present work judicious use of proxy allometric equations is explained and those for contributory parts of trees are derived for improvement in the allometric equations of large trees of *E. regnans*.

Supporting Table S1. Maximum DBH used for calibrating publicly accessible, species-specific carbon allometric equations, compared with maximum recorded tree diameters for some common TOF canopy species. DBH is indicative of gross sequestered carbon over lifetime, and of flutes in buttress. Allometric equations are generally available only for trees up to around half of maximum size.

| State habitat | TOF Species | Maximum DBH contributed to allometric (m) | Maximum DBH recorded (m) |
| --- | --- | --- | --- |
| TAS/VIC | *E. regnans* | 6.45 (Dean and Roxburgh 2006) | 10.76  (Ashton 1975) |
| VIC/TAS | *E. obliqua* (messmate/stringybark) | 3.50 (Keith *et al.* 2000) | 6.53 (McIntosh 2015) “Mt Cripps” TAS  6.31 (Maiden 1904) VIC |
| WA | *E. jacksonii* (red tingle) | None available | 5.92 (McIntosh 2015) “Pleated Lady” |
| VIC/TAS/NSW | *E. ovata syn E. gunnii var. acervula* (black gum) | 0.3 (Paul *et al.* 2013) | 5.82 (Maiden 1904) |
| VIC/TAS/NSW | *E. delegatensis* (gum-top-stringybark/alpine ash/white- top) | 0.83 (Keith *et al.* 2000) | 5.73 (McIntosh 2015) “Troll” TAS |
| WA | *E. diversicolor* (karri) | 1.51 (Grierson *et al.* 2000) | 4.14 (McIntosh 2015) “Hawke” |
| NSW/QLD | *E. pilularis* (blackbutt) | 1.29 (Applegate 1982) | 4.07 (Maiden 1917) |
| NSW/TAS/VIC | *E. viminalis* (manna gum) | 0.298 (Clifford *et al.* 2013) | 3.50 (McIntosh 2015) “White Knight” |
| WA | *Corymbia calophylla* (marri) | 1.10 (Grierson *et al.* 2000) | 3.44 (McIntosh 2015) “Poole” |
| WA | *E. marginata* (jarrah) | 1.84 (Grierson *et al.* 2000) | 3.28 (McIntosh 2015) “Hadfield” |
| NSW/QLD | *Syncarpia glomulifera*  (turpentine) | 0.70 (Eamus *et al.* 2000) | 2.91 (Maiden 1917) |
| NSW/QLD | *E. microcorys* (tallow-wood) | 0.90 (Eamus *et al.* 2000) | 2.72 (Maiden 1917) |

**The humus mounds of *E. regnans* in Tasmanian mixed-forest**

The mature eucalypts in primary mixed-forests have large and dynamic humus mounds, nestled in the buttress region. Humus mounds are more voluminous around the larger *E. regnans* trees because of high fall rates of branches and bark (trapping further debris), and high stemflow of rainwater. The widely spaced, mature eucalypt trees intercept oblique rain, from 35–70 m above the interstitial canopy of understorey trees. The consequently-high stemflow of rainwater is further concentrated by the large crown volume, steep branch-inclination angle ~45º (Supporting Figure S2), and smooth bark above the buttress (Crockford and Richardson 1990). The areas of increased stemflow infiltration are possible foci of microbial activity and microbial diversity (Levia *et al.* 2012). The humus mounds and hemi-epiphytes complicate measurement of the already complex buttress shape and lower trunks of the large trees, and therefore also of volume and carbon content of the host tree.

The Tasmanian forests support humus fires whereas they are absent in present-day Victoria (McCarthy *et al.* 1999). The difference between the remnant forest types in Tasmania and Victoria was noted by Ashton (1981) and linked to the likelihood of surface and humus-fuelled surface fires, and to soil substrate fertility. There is much less humus and fewer myrtles in present-day Victorian *E. regnans* forest (except near the Plenty and Ada Rivers where the 1939 fires did not penetrate and remove myrtles) (Ashton 1981; Ashton 1986). Additionally, there is an age difference: with fire being less frequent in Tasmania, many of the older stands in the Styx Valley of Tasmania are currently ~500 year old (Mount 1964; Wood *et al.* 2010) whereas the older mature forests in the VCH are generally only ~300 year old (Sillett *et al.* 2010).

The mature, larger *E. regnans* are often shorter and with wide buttresses, and have more open surroundings (Ashton 1975). This may be due to the stand being in the latter stages of stand thinning and the loss of gale protection from neighbours has made them lose their crowns but the open space has also allowed more photosynthesis with crown replacement and they have repeatedly lost and grown new upper branches over a few centuries, and thus increased in girth at the buttress-level. Larger buttress flutes increases the humus volume, unless there is critically advanced senescence with accompanying crown depletion, which will also reduce stemflow [of rainwater] and its associated SOC.


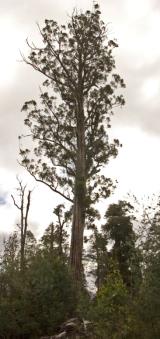

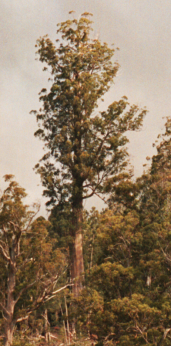

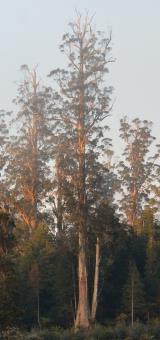


**A B C**


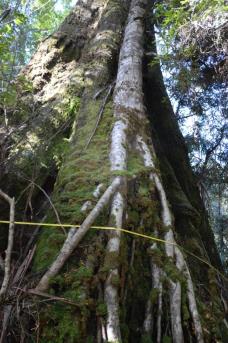

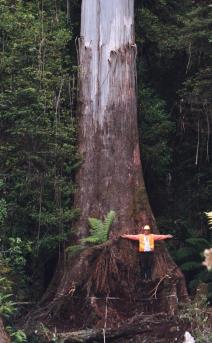


**D E**

Supporting Figure S2 (A), (B), and (C) Macroscopic aboveground mature *E. regnans* tree architecture. Acute angles, averaging near 45º, where large branches meet trunks. Epicormic shoots have become large branches, forming lower crowns following stand self-thinning, crown volume has increased ((A) Styx and (B) Florentine Valleys, both trees were extirpated by logging— cannot be re-measured). Tree ‘(b)’ was ‘El Grande’ DBH= 6.38 m, height= 75.4 m, photographed during logging. (C) large myrtle epiphyte on right-hand side, trees on edge of logging coupe SX009C. (D) Sapling sassafras as hemi-epiphyte on *E. regnans*, joint to 3.9 m height above soil A horizon, Styx Valley. Tree is ‘Chapel Tree’, DBH= 6.03, height= 80.1 m. (E) Most epiphytes cut away, person stood on epiphyte roots, prior to logging, DBH= 4.95 m, in logging coupe SX004C.

#### SUPPORTING METHODS

**
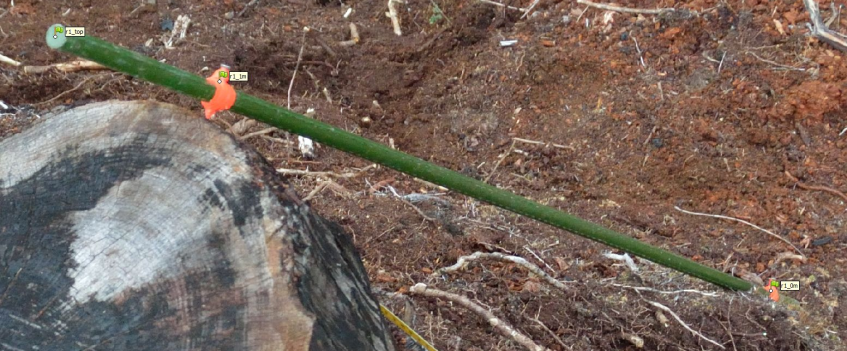
**

**A**

**
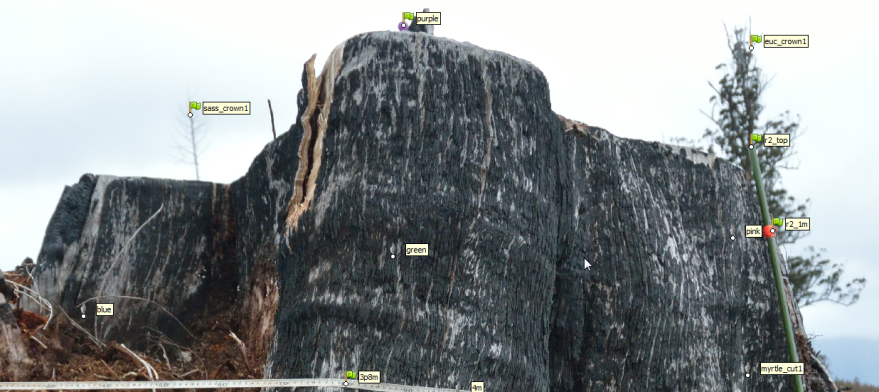
**

**B**

**
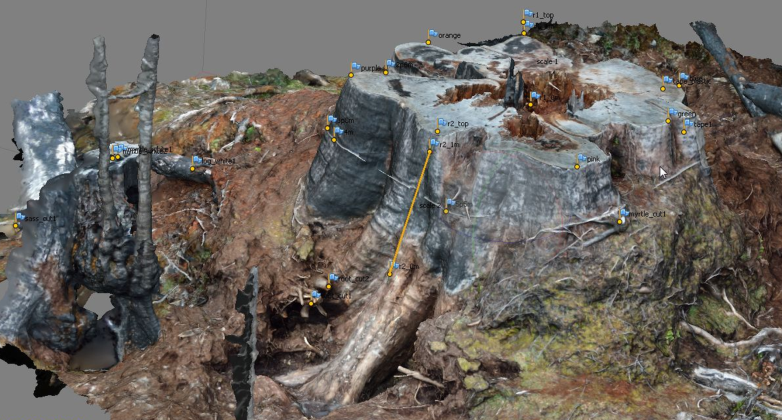
**

**C**

**Supporting Figure S3.** Example ground control points and tie points used. DBH= 3.11 m. (A) Placement of GCP at the intersection of screw and rod at top of scale bar, and tie point on end of rod. (B) Tie points on distant and near objects. (C) Completed 3D model with large number of tie points needed for object’s complexity, variety of backgrounds [in photos], and range of ground elevation from top to bottom.


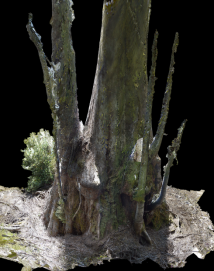

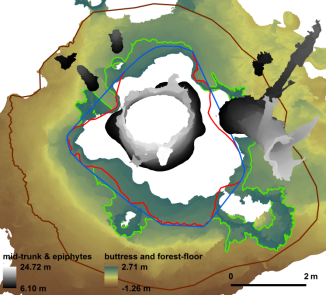


A B

Supporting Figure S4. Wider use of 3D models. Model of *E. regnans* DBH= 4.56 m (coupe SX009C), in Supporting Figure S2(C). The DBH could not be measured in the field due to the large hemi-epiphytic myrtle (LHS in (A), RHS in (B)). The myrtle could be separated in ArcGIS using cross-sections and contour levels, and the DBH then estimated, as shown in (b). (Green line= 1.3m contour, red line= corrected 1.3 m contour, blue line= 1.3 m convex hull, brown line= footprint). The lower, oblique view of the model is as viewed from uphill in the top view.


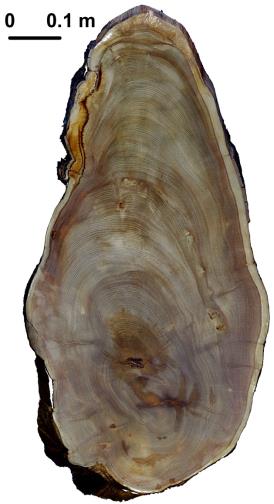
Supporting Figure S5.

(A) Orthophoto created using terrestrial photography and Photoscan of a *Eucalyptus regnans*, (DBH 4.38 m, Tyenna Valley, coupe TN050E) root slice of large lateral within the footprint, ring age count= 350(±40) years: most expansive growth on top side, corresponding to buttress width expansion. (B) GoogleEarth^®^ satellite image shows felled trunk, stump, and neighbouring stumps, during logging (scale bar = 40 m).

**A**


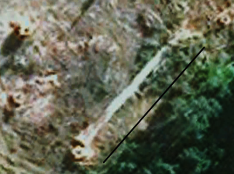


**B**


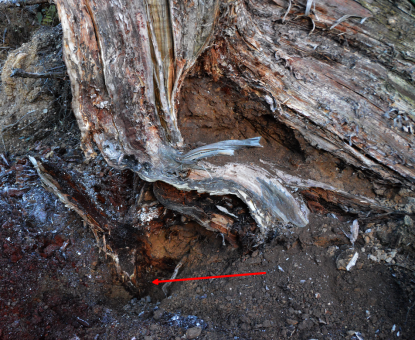


**A**


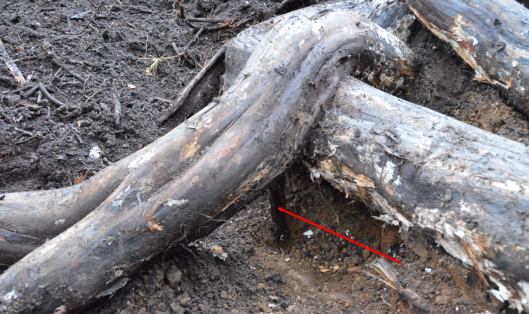


**B**

Supporting Figure S6. Sinker roots on mature *E. regnans*. (A) Large sinker root beneath edge of buttress and adjoining the large lateral (right-hand side of photograph). The area around the tree had been logged (coupe SX009C) and the tree had fallen and its buttress split open. (B) The tree in coupe TN050E had been logged. Soil from the coarse roots on one buttress spur was removed. Small sinker roots, not part of the buttress, were within the footprint zone.

**Supporting Literature cited**

ANM. 1979. *Working Plan For Concession Area Of Australian Newsprint Mills Limited In The Derwent Valley*, Boyer, Tasmania: Australian Newsprint Mills Limited, Boyer.

ANM. c1960. *Australian Newsprint Mills. Unpublished Coupe Registers, Florentine, Tyenna and Styx Valleys*, Forestry Tasmania Library, Hobart, Tasmania.

Applegate GB. 1982. Biomass of Blackbutt (*Eucalyptus pilularis* Sm.) forests on Fraser Island. Masters, University of New England, University of New England.

Ashton DH. 1975. The root and shoot development of *Eucalyptus regnans* F. Muell. *Australian Journal of Botany* 23:867-887.

Ashton DH. 1981. Fire in tall open-forests (wet sclerophyll forests). In: Gill AM, Groves RH, Noble IR, eds. *Fire and the Australian Biota*. Canberra: Australian Academy of Science, 339-366.

Ashton DH. 1986. Ecology of bryophytic communities in mature *Eucalyptus regnans* F. Muell. forest at Wallaby Creek, Victoria. *Australian journal of Botany* 34:107-129.

Ashton DH, Attiwill PM. 1994. Tall Open-forests. In: Groves RH, ed. *Australian Vegetation*. Cambridge: Cambridge University Press, 157-196.

Beilin R. 2007. Landscape with voices: reflecting on resilience on farms in the ‘Heartbreak Hills’, Strzelecki ranges. *Local-Global* 4:141-160.

Bowman DMJS, Kirkpatrick JB. 1984. Geographic variation in the demographic structure of stands of *Eucalyptus delegatensis* R. T. Baker on dolerite in Tasmania. *Journal of Biogeography* 11:427-437.

Bowman DMJS, Williamson GJ, Keenan RJ, Prior LD. 2014. A warmer world will reduce tree growth in evergreen broadleaf forests: evidence from Australian temperate and subtropical eucalypt forests. *Global Ecology and Biogeography* 23:925–934.

Brown S. 1997. *Estimating biomass and biomass change of tropical forests: a primer. UN FAO Forestry Paper 134*, Rome: FAO.

Chave J, Andalo C, Brown S, Cairns MA, Chambers JQ, Eamus D, Folster H, Fromard F, Higuchi N, Kira T, Lescure JP, Nelson BW, Ogawa H, Puig H, Riera B, Yamakura T. 2005. Tree allometry and improved estimation of carbon stocks and balance in tropical forests. *Oecologia* 145:87-99.

Clifford D, Cressie N, England JR, Roxburgh SH, Paul KI. 2013. Correction factors for unbiased, efficient estimation and prediction of biomass from log-log allometric models. http://ro.uow.edu.au/cgi/viewcontent.cgi?article=1122&context=cssmwp (09-September-2015).

Cremer KW. 1960. Eucalypts in rain forest. *Australian Forestry* 24:120-126.

Crockford RH, Richardson DP. 1990. Partitioning of rainfall in a eucalypt forest and pine plantation in southeastern Australia: II Stemflow and factors affecting stemflow in a dry sclerophyll eucalypt forest and a *Pinus radiata* plantation. *Hydrological Processes* 4:145-155.

Dean C. 2003. Calculation of wood volume and stem taper using terrestrial single-image close-range photogrammetry and contemporary software tools. *Silva Fennica* 37:359-380.

Dean C, Roxburgh SH. 2006. Improving visualisation of mature, high-carbon-sequestering forests. *Forest Biometry, Modelling and Information Sciences* 1:48-69.

Dean C, Roxburgh SH, Mackey BG. 2003. Growth Modelling of *Eucalyptus regnans* for Carbon Accounting at the Landscape Scale. In: Amaro A, Reed D, Soares P, eds. *Modelling Forest Systems*. Wallingford, Oxford, U.K.: CABI Publishing, 27-39 + plates.

Dean C, Wardell-Johnson GW, Kirkpatrick JB. 2012. Are there any circumstances in which logging primary wet-eucalypt forest will not add to the global carbon burden? *Agricultural and Forest Meteorology* 161:156-169.

Dietrich P. 2012. Carbon stocks in coarse woody debris and soil in the mixed forests and the rainforests in southern Tasmania. MSc, Technische Universität, Dresden, Germany.

Eamus D, McGuinness K, Burrows W. 2000. *Review of Allometric Relationships for Estimating Woody Biomass for Queensland, the Northern Territory and Western Australia. National Carbon Accounting System Technical Report No. 5A*, Canberra: The Australian Greenhouse Office.

Fedrigo M, Kasel S, Bennett LT, Roxburgh SH, Nitschke CR. 2014. Carbon stocks in temperate forests of south-eastern Australia reflect large tree distribution and edaphic conditions. *Forest Ecology and Management* 334:129-143.

Ferguson KVM. 1948. Some Statistics of Timber Yields from Virgin Stands of White Mountain Ash. *Australian Forestry* 12:13-15.

Fox-Hughes P, Harris R, Lee G, Grose M, Bindoff N. 2014. Future fire danger climatology for Tasmania, Australia, using a dynamically downscaled regional climate model. *International Journal of Wildland Fire* 23:309–321.

Gilbert JM. 1959. Forest succession in the Florentine Valley, Tasmania. *papers and Proceedings of the Royal Society of Tasmania* 93:129-151.

Grierson P, Williams K, Adams M. 2000. *Review of Unpublished Biomassrelated Information: Western Australia, South Australia, New South Wales and Queensland. National Carbon Accounting System Technical Report No. 25*, Canberra: Commonwealth of Australia.

Harmon ME. 2001. Carbon sequestration in forests. Addressing the scale question. *Journal of Forestry* 99:24-29.

Harmon ME. 2009. Woody Detritus Mass and its Contribution to Carbon Dynamics of Old-Growth Forests: the Temporal Context. In: C. W, G. G, M. H, eds. *Old-growth Forests: Function, Fate and Value. Ecological studies*. New York: Springer-Verlag, 159-190.

Henry M, Jara MC, Réjou-Méchain M, Piotto D, Fuentes JMM, Wayson C, Guier FA, Lombis HC, López EC, Lara RC, Rojas KC, Pasquel JDÁ, Montoya ÁD, Vega JF, Galo AJ, López OR, Marklund LG, Milla F, Cahidez JdJN, Malavassi EO, Pérez J, Zea CR, García LR, Pons RR, Sanquetta C, Scott C, Westfall J, Zapata-Cuartas M, Saint-André L. 2015. Recommendations for the use of tree models to estimate national forest biomass and assess their uncertainty. *Annals of Forest Science* 72:769–777.

Herrmann W. 2006. Vulnerability of Tasmanian giant trees. *Australian Forestry* 69:285–298.

Hickey J, Davis S, Wardman R, Harris J. 1993. How much rainforest is in Tasmania? A better answer to a difficult question. *Tasforests* 5:13-24.

IPCC. 2003. IPCC Good practice guidance for land use, land-use change and forestry. In: Penman J, Gytarsky M, Hirashi T, Krug T, Kruger D, Pipatti R, Buendia L, Miwa K, Ngara T, Tanabe K, Wagner F, eds. Hayama, Kanagawa, Japan: National Greenhouse Gas Inventories Programme Technical Support Unit, Institute for Global Environmental Strategies.

Keith H, Barrett D, Keenan R. 2000. Review of allometric relationships for estimating woody biomass for New South Wales, the Australian Capital Territory, Victoria, Tasmania and South Australia. http://pandora.nla.gov.au/pan/23322/20020220-0000/www.greenhouse.gov.au/ncas/files/pdfs/tr05bfinal.pdf (20-May-2016).

Keith H, Mackey BG, Lindenmayer DB. 2009. Re-evaluation of forest biomass carbon stocks and lessons from the world’s most carbon-dense forests. *Proceedings of the National Academy of Sciences of the United States of America* 106:11635-11640.

Kirkpatrick JB. 1986. Some ecological aspects of forest conservation in temperate Australia. In: Hanxi Y, Zhan W, Jeffers JNR, Ward PA, eds. *International Symposium on Temperate Forest Ecosystem Management and Environmental Protection*. Changbai Mountain Research Station Academia Sinica Antu, Jilin Province, People's Republic of China: Institute of Terrestrial Ecology, Natural Environment Research Council, 68-77.

Kirkpatrick JB. 1994. *A Continent Transformed. Human Impact on the Natural Vegetation of Australia*, Melbourne: Oxford University Press.

Kirkpatrick JB, DellaSala DA. 2011. Temperate Rainforests of Australasia. In: DellaSala DA, ed. *Temperate and Boreal Rainforests of the World: Ecology and Conservation*. Washington DC: Island Press, 195-212.

Levia DF, Van Stan IIJT, Inamdar SP, Jarvis MT, Mitchell MJ, Mage SM, Scheick CE, McHale PJ. 2012. Stemflow and dissolved organic carbon cycling: temporal variability in concentration, flux, and UV-Vis spectral metrics in a temperate broadleaved deciduous forest in the eastern United States. *Canadian Journal of Forest Research* 42:207-216.

Lindenmayer DB, Laurance WF, Franklin JF. 2012. Global decline in large old trees. *Science* 1305.

Lindenmayer DB, Hunter ML, Burton PJ, Gibbons P. 2009. Effects of logging on fire regimes in moist forests. *Conservation Letters* 2:271-277.

Mace B. 1996. Mueller— Champion of Victoria's giant trees. *Victorian Naturalist* 113:198-207.

Mackey BG, Keith H, Berry SL, Lindenmayer DB. 2008. *Green Carbon. The Role of Natural Forests in Carbon Storage. Part 1: A Green Carbon Account of Australia’s South-eastern Eucalypt Forests, and Policy Implications*, Canberra: Australian National University E Press.

Maiden JH. 1904. Where are the Largest Trees in the World. In: *The Sydney Morning Herald*. Sydney: 3.

Maiden JH. 1917. *Forestry Handbook. Part Il. Some of the Principal Commercial Trees of New South Wales*, Sydney: Forest Department of New South Wales.

Mainville DM. 2007. The Impacts of Agriculture and Plantation Forestry in a Selection of Upper Catchments of the Strzelecki Ranges, Victoria. RMIT University, Melbourne.

McCarthy MA, Gill MA, Lindenmayer DB. 1999. Fire regimes in mountain ash forest: evidence from forest age structure, extinction models and wildlife habitat. *Forest Ecology and Management* 124:193-203.

McIntosh D. 2015. National Register of Big Trees. Australia's Champion Trees. http://www.nationalregisterofbigtrees.com.au/ (09-September-2015).

Melson SL, Harmon ME, Fried JS, Domingo JB. 2011. Estimates of live-tree carbon stores in the Pacific Northwest are sensitive to model selection. *Carbon Balance and Management* 6:1-16.

Moroni MT, Kelley TH, McLarin ML. 2010. Carbon in Trees in Tasmanian State Forest. *International Journal of Forestry Research* 2010.

Mount AB. 1964. Three Studies in Forest Ecology. University of Tasmania.

Nabuurs GJ, Schelhaas MJ. 2002. Carbon profiles of typical forest types across Europe assessed with CO2FIX. *Ecological Indicators* 1:213-223.

Ngomanda A, Mavouroulou QM, Obiang NLE, Iponga DM, Mavoungou JF, Lépengué N, Picard N, Mbatchi B. 2012. Derivation of diameter measurements for buttressed trees, an example from Gabon. *Journal of Tropical Ecology* 28:1-4.

Nogueira EM, Nelson BW, Fearnside PM. 2006. Volume and biomass of trees in central Amazonia: influence of irregularly shaped and hollow trunks. *Forest Ecology and Management* 227:14-21.

Paul KI, Roxburgh SH, England JR, Ritson P, Hobbs T, Brooksbank K, Raison J, Larmour JS, Murphy S, Norris J, Neumann C, Lewis T, Jonson J, Carter JL, McArthur G, Barton C, Rose B. 2013. Development and testing of allometric equations for estimating above-ground biomass of mixed-species environmental plantings. *Forest Ecology and Management* 310:483–494.

Petrie AHK, Jarrett PH, Patton RT. 1929. The vegetation of the Blacks' Spur region: A study in the ecology of some Australian mountain eucalyptus forests: I. The mature plant communities. *Journal of Ecology* 17:223-248.

Roxburgh SH, Wood SW, Mackey BG, Woldendorp P, Gibbons P. 2006. Assessing the carbon sequestration potential of managed forests: a case study from temperate Australia. *Journal of Applied Ecology* 43:1149-1159.

Sillett SC, Van Pelt R, Kramer RD, Carroll AL, Koch GW. 2015. Biomass and growth potential of *Eucalyptus regnans* up to 100 m tall. *Forest Ecology and Management* 348:78-91.

Sillett SC, Van Pelt R, Koch GW, Ambrose AR, Carroll AL, Antoine ME, Mifsud BM. 2010. Increasing wood production through old age in tall trees. *Forest Ecology and Management* 259:976-994.

Singh N, Abiven S, Torn MS, Schmidt1 MWI. 2012. Fire-derived organic carbon in soil turns over on a centennial scale. *Biogeosciences* 9:2847–2857.

The Mercury. 1941. How plan evolved round Tasmanian forest giants. In: *The Mercury*. Hobart, Tasmania: 6-7.

Turner PAM, Balmer J, Kirkpatrick JB. 2009. Stand-replacing wildfires? The incidence of multicohort and single-cohort Eucalyptus regnans and E. obliqua forests in southern Tasmania. *Forest Ecology and Management* 258:366-375.

Wood SW, Hua Q, Allen KJ, Bowman DJMS. 2010. Age and growth of a fire prone Tasmanian temperate old-growth forest stand dominated by *Eucalyptus regnans*, the world's tallest angiosperm. *Forest Ecology and Management* 260:438-447.

WRI. 2010. Sawlog and Pulpwood Markets 4Q/09 - Asia & Oceania. *Wood Resource Quarterly* 4Q/2009:36-47.

WRI. 2014. Downward trend in Australian chip exports broken; China surpassed Japan as the major destination in the 1Q/14. *Wood Resources Quarterly* 1Q/2014.

Zimmermann M, Bird MI, Wurster C, Saiz G, Ck IGI, Barta JIRI, Capek P, Santruckova H, Smernik R. 2012. Rapid degradation of pyrogenic carbon. *Global Change Biology* 18:3306-3316.

# 
